# Genetic correlation network prediction of forest soil microbial functional organization

**DOI:** 10.1038/s41396-018-0232-8

**Published:** 2018-07-25

**Authors:** Bin Ma, Kankan Zhao, Xiaofei Lv, Weiqin Su, Zhongmin Dai, Jack A. Gilbert, Philip C. Brookes, Karoline Faust, Jianming Xu

**Affiliations:** 1 Institute of Soil and Water Resources and Environmental Science, College of Environmental and Resource Sciences, Zhejiang University, Hangzhou, 310058 China; 2 Zhejiang Provincial Key Laboratory of Agricultural Resources and Environment, Hangzhou, 310058 China; 3 The Microbiome Center, Department of Surgery, University of Chicago, Chicago, IL 60637 USA; 4 Bioscience Division, Argonne National Laboratory, Lemont, IL 60439 USA; 5 Department of Microbiology and Immunology, Rega Institute, KU Leuven, Campus Gasthuisberg, Leuven, Belgium

## Abstract

Soil ecological functions are largely determined by the activities of soil microorganisms, which, in turn, are regulated by relevant interactions between genes and their corresponding pathways. Therefore, the genetic network can theoretically elucidate the functional organization that supports complex microbial community functions, although this has not been previously attempted. We generated a genetic correlation network based on 5421 genes derived from metagenomes of forest soils, identifying 7191 positive and 123 negative correlation relationships. This network consisted of 27 clusters enriched with sets of genes within specific functions, represented with corresponding cluster hubs. The clusters revealed a hierarchical architecture, reflecting the functional organization in the soil metagenomes. Positive correlations mapped functional associations, whereas negative correlations often mapped regulatory processes. The potential functions of uncharacterized genes were predicted based on the functions of located clusters. The global genetic correlation network highlights the functional organization in soil metagenomes and provides a resource for predicting gene functions. We anticipate that the genetic correlation network may be exploited to comprehensively decipher soil microbial community functions.

## Introduction

Microorganisms operate at the heart of biological characteristics, biogeochemical processes, and ecology of soils [1]. However, elucidating the microbial functions that underpin these properties of soils can be challenging, primarily due to the numerical abundance of microbes [2] and their vast taxonomic and functional diversity [3] in the soil environment, which contains extreme spatial heterogeneity and complex chemical and biological properties [4]. A gram of soil contains an average of 10^9^ prokaryotic cells, and ~10^5^ distinct prokaryotic genomes [5]. We estimate that the majority of soil microbial genomes have yet to be sequenced [6], as such we have limited understanding of the link between soil microbial community composition and functionality, which is complicated by the horizontal gene transfer promiscuity of some bacterial lineages [7]. However, metagenomic sequencing can provide a snapshot of the relative abundance of genes and genotypes, providing an opportunity to glimpse soil microbial functional potential [8–11].

Ecosystems are formed by the hierarchical organization from populations, individuals, pathways, and genes to communities [12]. All the macroscopic properties such as community functions are depended on how the microscopic building blocks (genes, genotypes, and cells) are assembled and interact [13]. Genetic interactions at the cellular scale have long been investigated in model organisms, especially in yeast, for identifying functional relationships between genes [14–16], whereby their interactions imply that two genes share a functional relationship [17]. Studies exploring these interactions have identified many biological functions, including functional dependency and redundancy, which are governed by the interactions between several enzymes, [18]. Although only ~1000 core genes in the yeast genome are lethal when mutated, there are around 550,000 synthetic lethal genetic interaction pairs, including an extreme set of ~10,000 genetic interactions between non-core genes [16]. However, the complexity of genetic interactions in an assemblage at the community scale has not yet been evaluated.

Microbial genetics represents a unique platform to determine whether genetic interaction modeling can be used to elucidate relevant interactions between genes. Conservation of central metabolic functional genes [19] and high redundancy of functional genes in microbial communities [20] suggest that genomes of different taxa might contain similar functional genes. Therefore, metagenomic datasets can display stable genetic interaction patterns because similar functional genes from different taxa might have a similar response to environmental variations. With metagenomics, genetic interactions cannot be quantified using the combinatorial construction of mutants but can be explored using the pairwise correlation coefficient [21, 22]. A matrix of gene-gene pairwise correlations can therefore be used to systematically predict potential genetic interactions among genetic elements in a database.

Network analysis has facilitated many discoveries in both systems biology and microbial ecology [23], providing a platform on which to determine relationships between data that can predict genetic interactions [15, 16], protein–protein interactions [24, 25], and metabolic reactions [22, 26]. Network analysis has also been applied to evaluate microbial community assemblies in soil [27, 28] and rhizosphere microbiomes [29]. However, network analysis of soil metagenomic correlation patterns has not previously been explored.

Here we employed genetic correlation network analysis using gene abundances from a database of 45 soil metagenomes from eastern China (Fig. S1). To better capture the high functional redundancy of microbial communities and reduce bias in correlation coefficients induced by sparse data, we filtered non-core genes occurring in only few samples and focus on core genes. Heuristic clustering approaches were employed to examine the intrinsic associations between functional groups, and hub genes were identified for each cluster. Each cluster was found to represent different potential functionalities, and a hierarchical structure was observed with different topologies at different resolutions of functional annotation. Functional predictions based on genetic interactions were made for genes of unknown function, which were validated with structural predictions. This investigation represents a significant advance in soil microbiome systems biology.

## Materials and methods

### Sample collection

We collected three soil samples from a 100 × 100 m^2^ plot at each of the 45 sites across five continual vegetation types in Eastern China (Fig. S1) using a uniform sampling protocol. Samples were collected at a depth of 0–15 cm, after the removal of loose debris from the forest floor. Five soil cores were combined to obtain one soil sample, resulting in three analytical sample replicates per plot. All soil samples were transported to the laboratory on ice. Coarse roots and stones were removed, and a subset of the soil was air-dried for analysis of edaphic properties. Methods to obtain values for all measured edaphic variables are described in a previous study [27].

### DNA extraction and sequencing

Upon arrival at the laboratory, DNA was extracted from fresh soil samples using the MP FastDNA SPIN Kit for soil (MP Biomedicals, LLC, Ohio, USA) as per manufacturer’s instructions. Equal concentrations (200 µg) of DNA extract from the three replicates were combined to form a composite genetic pool representing total DNA for each site. DNA purity and concentration were determined using a NanoDrop spectrophotometer (NanoDrop Technologies Inc., Wilmington, DE, USA). Isolated total DNA was stored at −20 °C for microbial diversity and sequence analyses. Shotgun sequencing of metagenomic DNA on an Illumina HiSeq 2500 platform (Illumina, Inc., San Diego, CA, USA) at the Novogene (Tianjin, China) produced a total of ~1.5 billion paired-end reads (read length = 150 bp). About 93.1% of reads were above Q30. As far as we known, this is the biggest forest soil metagenome shotgun data set to date. Sequence data have been deposited in the public National Center for Biotechnology Information (NCBI) database under BioProject accession number PRJNA475650.

### De novo genomic assembly and annotation

Raw shotgun sequencing reads were preprocessed using ngsShoRT v2.1 [30] with lqr_5adpt_tera method (Table S7). Whole genome de novo assemblies for each sample were performed using IDBA-UD [31] with the following parameters: -mink 50, -maxk 92, -step 4, -min_contig 500. Contigs from all samples were combined and reassembled with minimum 2. The quality of assemblies was evaluated using MetaQUAST v2.2 [32]. The contigs were assigned to known bacterial genomes from RefSeq using CLARK [33]. Paired-end sequencing reads were mapped to assembled contigs using BWA v0.7.16a [34] to generate read coverage information for assembled contigs. The mapped read counts were extracted using SAMtools v1.4 [35]. Open reading frame prediction and annotation were performed using Prodigal v2.50 [36]. The resulting protein translations were assigned by comparisons to Pfam 31.0 [37] using HMMER 3 [38], to KEGG release 84.0 using GhostKOALA [39], and to Uniprot database using BLASTP (best hit with *E* < 0.001). The entities were manually annotated to related GO terms using quickGO tool [40]. The KO numbers were mapped on wiring diagrams of a metabolic pathway map using KEGG mapping (Fig. S8). Except for typical metazoan pathways (hormones, bile acids), most functions of the global KEGG map are represented in the forest soil, which underlines the high functional diversity of soil. The taxonomic classification of contigs was performed with CLARK (V1.2.4) [33].

### Network construction and analysis

A Spearman correlation matrix among genes was calculated based on the relative abundance of genes in each sample. To reduce the bias of correlation coefficients induced by sparse gene matrix, only genes detected in 25 out of 45 samples were used for network construction. The indirect connections were reduced with the deconvolution method (*α* = 1, *β* = 0.99) [41]. Random matrix theory (RMT) was used to automatically identify the appropriate similarity threshold prior to network construction [42]. The connections in the network represents the positive or negative correlation values greater than the threshold value (determined with RMT) and the *P* values of correlation (adjusted with false discovery rate method [43]) smaller than 0.05. Network properties were characterized with the *igraph* package [44] and networks were graphed using Gephi [45]. We defined genes presenting in all samples as core genes, and genes presenting in 25 to 44 samples as non-core genes. An Erdős-Rényi network with the same number of vertices and edges was generated with *erdos.renyi.game* function in igraph.

Clusters were unfolded using the heuristic method at various resolution values [46]. The nodes with the highest connection numbers (ranging from 16 to 60) in each cluster were defined as hub nodes, and the other nodes were defined as peripheral nodes. The connectivity of nodes was determined based on their within-cluster connectivity and between cluster connectivity. The connectivity was used to classify the nodes based on the topological roles they play in the network. The nodes without between cluster connectivity were intra-cluster nodes; the nodes with between cluster connectivity were inter-cluster nodes.

The hierarchical structure of clusters was identified based on the combination pattern of clusters at resolution 1–15. The parent nodes in the hierarchical tree represents the parent clusters combined from sibling clusters of lower resolution levels. The connectivity between clusters was defined as the total connection number between all the nodes from different clusters. Given that the degree is non-normally distributed, the negative connection numbers were compared with the Wilcoxon rank sum test. To validate the predicted functions for genes with domains of unknown functions (*DUF*), the structure of protein domains in DUF genes were modeled by homology modeling with SWISS-MODEL.

## Results

### A global genetic correlation network in metagenomes of pristine forest soils

**Fig. 1:**
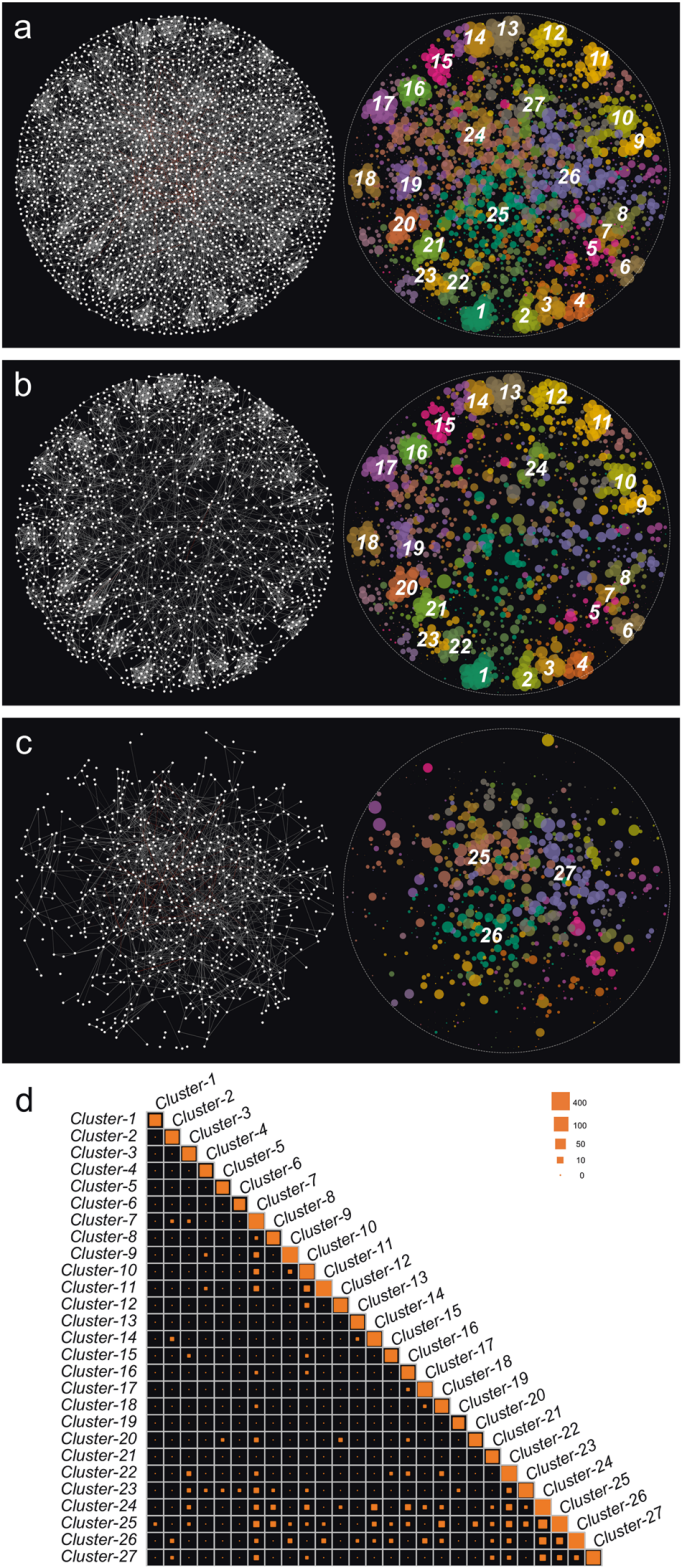
A network of genetic correlation relationships. **a** A global genetic correlation network encompassing all core and non-core genes was constructed from the genetic correlation matrix. Gene pairs with Spearman coefficients > 0.818 were connected and graphed using a force-directed layout algorithm. Genes with high correlation coefficients map were proximal to each other, whereas genes with low correlation coefficients were positioned further apart. The clusters in the global network were detected with a multi-level aggregation method. Twenty-seven dominant clusters are represented with different colors. **b** A genetic correlation subnetwork for core genes, which dominated in 24 clusters. **c** A genetic correlation subnetwork for non-core genes, which dominated in three clusters. **d** Connection frequency within and between clusters. Tile size reflects the connections frequency observed for a given pair of clusters in the global genetic correlation network. Tiles on the diagonal represent the frequency of connections among genes belonging to the same cluster. Tiles off the diagonal represent the frequency of connections between different clusters

A genetic correlation network was constructed from a Spearman’s Rank correlation coefficient matrix of the relative abundance of 5421 genes annotated from the 45 soil metagenomes. Genetic correlation connections linked 2641 gene nodes through 7314 connections, corresponding to 7191 positive and 123 negative correlations (Fig. 1a). By comparing the binomial degree distribution in randomly linked Erdős-Renyi networks with the same numbers of nodes and edges, the power-law degree distribution (Fig. S2) and non-random distribution of negative correlations (Fig. 1a–c) suggests that the derived network is non-random and scale-free (*R*^2^ = 0.83 in log-log scale). The subnetwork for core genes (genes found in all of the 45 metagenomes) comprised 1635 nodes and 4135 connections (Fig. 1b), whereas the subnetwork for non-core genes comprised 826 nodes and 1,374 connections (Fig. 1c). In total, 1805 connections occurred between core and non-core genes. Although the network density values were similar in the subnetworks for core and non-core genes, the clustering coefficient of the core gene subnetwork (Fig. 1b) was twofold greater than that of the subnetwork for non-core genes (Fig. 1c) (Table S1). The diameter of the core gene subnetwork was larger than in the global network (Table S1).

A heuristic method was used to detect the network clusters, in which the genes displayed similar connection profiles. We detected 27 dominant clusters, which contained 1704 intensity wired nodes. The remaining 757 nodes, which did not belong to these clusters, were treated as loose wired nodes. Among the 27 clusters in the global genetic correlation network, 24 clusters were dominated by core genes (Fig. 1b) and three clusters were dominated by non-core genes (Fig. 1c). The connection intensity in the 23 clusters, localized at the periphery of the network layout (cluster 1–23), was greater than in the four clusters localized at the center (Fig. 1a). The majority of connections associated with the 27 clusters were intra-cluster connections that linked nodes from the same clusters (Fig. 1d, on-diagonal). The inter-cluster connections that link different clusters were mainly found in clusters 24–26 (Fig. 1d, off-diagonal).

**Fig. 2:**
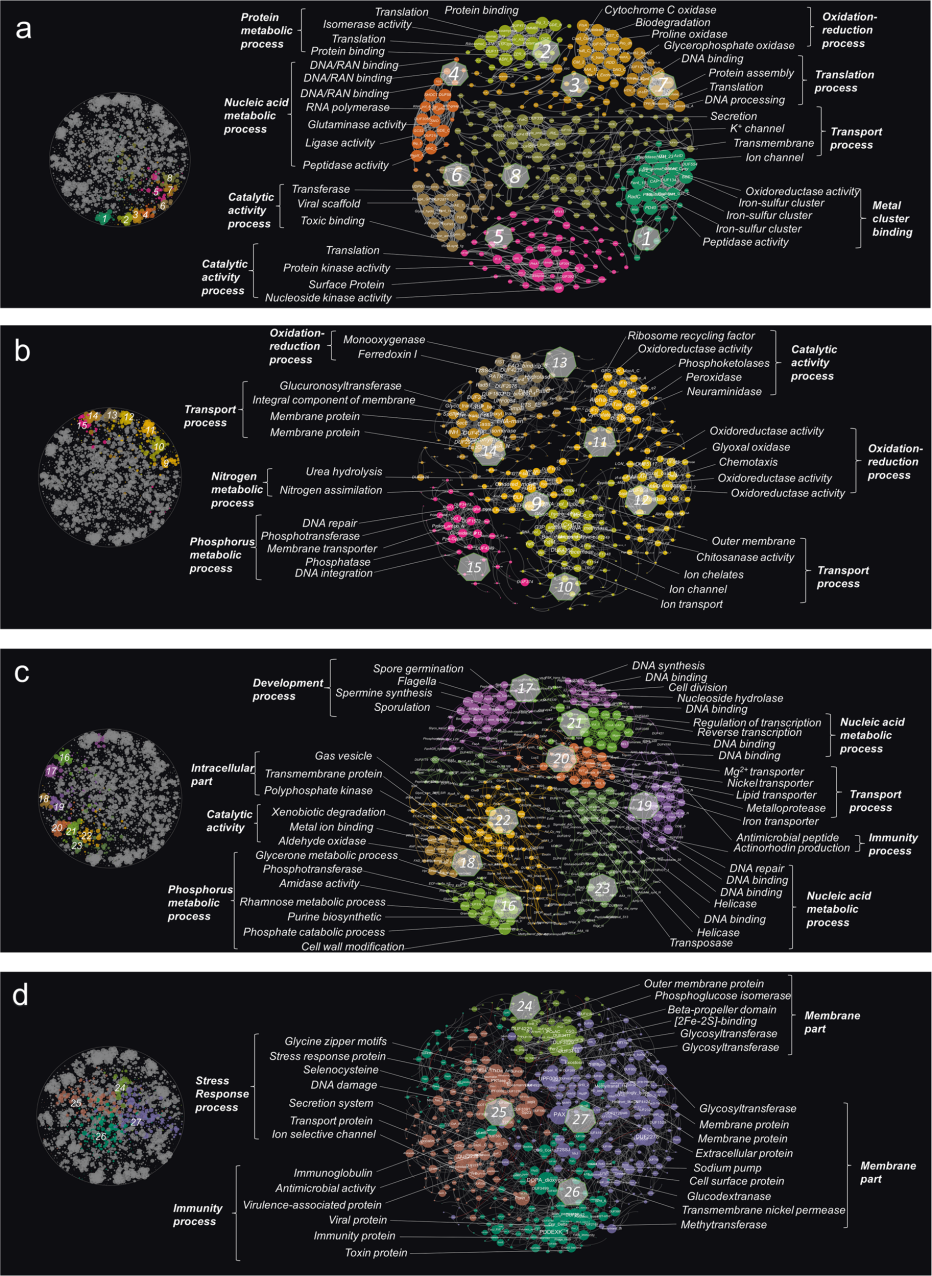
Clusters in the genetic correlation network. **a** Genes localized within the cluster 1–8. **b** Genes localized within the cluster 9–15. **c** Genes localized within the cluster 16–23. **d** Genes localized within the cluster 24–27

Functional relationships associated within clusters were resolved in greater detail by extracting clusters from the global network and visualizing them in groups (Fig. 2). Genes related to similar functions tended to co-associate in the same clusters. The core gene subnetwork (Fig. 1b) comprised clusters related to protein and nucleic acid metabolic processes, nutrient utilization, immunity, oxidation/reduction, and catalytic processes (Fig. 2a–d), whereas the non-core gene subnetwork comprised clusters enriched for genes related to the stress response, immunity, and membrane structure (Fig. 2d). Different clusters were associated with various taxonomic profiles (Fig. S4). Although the cluster number per genus was not linearly correlated with the abundance of genera (Fig. S5A), the abundances of the generalists (cluster per genus ≥ 20) were significantly higher than the abundances of the specialists (cluster per genus ≤ 5, Tukey-HSD, *P* = 0.05) and the moderate general genus (6 ≥ cluster per genus ≥ 19, Tukey-HSD, *P* = 0.04) (Fig. S5B). In the generalist enriched phylum Firmicutes and specialist enriched phylum Bacteroidetes (Fig. S5C), the genus *Capnocytophaga* only contributed to the transport process (cluster 10) and nucleic acid metabolic process (cluster 23), and the genus *Rhodothermus* only contributed to the nitrogen metabolic process (cluster 9), transport process (cluster 11), catalytic activity process (cluster 12), and immunity process (cluster 26) (Fig. S4).

The environmental factors that significantly influenced both the profiles of genera (Fig. S6A) and genes (Fig. S6B) of soil communities, including longitude, latitude, temperature, precipitation, and dissolved Fe and Al, possessed the greatest number of links to genes (Fig. S7). Longitude, latitude, temperature, precipitation, and soil pH influenced similar gene sets and most of these genes were not correlated with other genes and hence were not involved in the genetic network (Fig. S6). Available K, soil C/N ratio, and dissolved Al and Fe influenced the largest number of genes involved in the genetic correlation network (Table S3). Although total and dissolved nitrogen correlated with a small number of genes, their connections were specifically linked with cluster 9, which was annotated as the gene cluster for nitrogen utilization. The links for the humic acid/fulvic acid ratio were specifically connected with cluster 20, which was annotated as the gene cluster for transport processes.

### Cluster hubs of the genetic correlation network

**Fig. 3:**
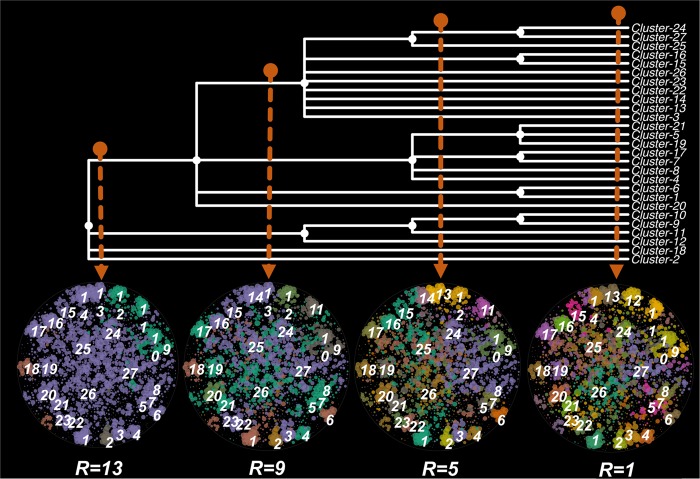
The hierarchy of clusters in the global genetic correlation network at different resolution (R) of modularity. Lower resolution detects smaller communities and higher than 1.0 larger ones. Distinct sibling clusters resolved at one resolution level of the hierarchical level combined together at a higher level to generate a larger parent cluster, which indicates closely related functions among its sibling clusters

In total, we identified 59 cluster hub genes (those that possessed the greatest number of connections in each cluster) from the 27 dominant clusters (Fig. 3a). We measured the intra-cluster and inter-cluster connections of genes based on the within and between cluster edge numbers. An inter-cluster gene involved in diverse functions was expected to possess connections between clusters (between cluster edge number > 0), and intra-cluster genes were expected to connect with nodes within the same cluster (between cluster edge number = 0). We identified 22 hub genes as inter-cluster genes and 37 hub genes as intra-cluster genes (Fig. 3a). Among these hub genes, 48 were core genes and 11 were non-core genes (Fig. S3). The core hub genes were mainly localized in the clusters dominated with core genes (cluster 1–23), whereas the non-core hub genes were mainly localized in the clusters dominated with non-core genes (cluster 24–27). Similarly, the intra-cluster hub genes were mainly found in clusters dominated with core genes, and the inter-cluster hub genes were mainly localized in the clusters dominated with non-core genes (Fig. 3b). The intra-cluster hub genes were densely connected with the genes within the corresponding clusters (Fig. 3b) and associated with the functions of the corresponding clusters (Table S2). For example, (i) the intra-cluster hub genes *RadC*, *Hydrolase*, and *Pro-kuma-activ*, encoding a domain of hydrolase, were the hubs for clusters associated with oxidation-reduction processes or catalytic activities; (ii) the membrane protein gene *MgtC* was the hub for the cluster associated with transport process; (iii) the binding protein gene *SHOCT* was the hub for the cluster associated with metal cluster binding; and (iv) the polyphosphate kinase gene (*Ppx*) was the hub for the cluster associated with phosphate metabolism (Table S2). In contrast, the inter-cluster hub genes provided clues for the association between clusters (Fig. 3b). For example, the binding protein genes *HTH_18* and *PrlF_antitoxin* served as articulation among clusters for transport processes (cluster 20), nucleic acid metabolism (cluster 21 and 23), and intracellular parts (cluster 22). The outer membrane protein gene *OmpH* articulated between the cluster for transport processes (cluster 10), nitrogen utilization (cluster 9), and catalytic processes (cluster 11) (Fig. 3b).

### Hierarchy of the clusters in the genetic correlation network

**Fig. 4:**
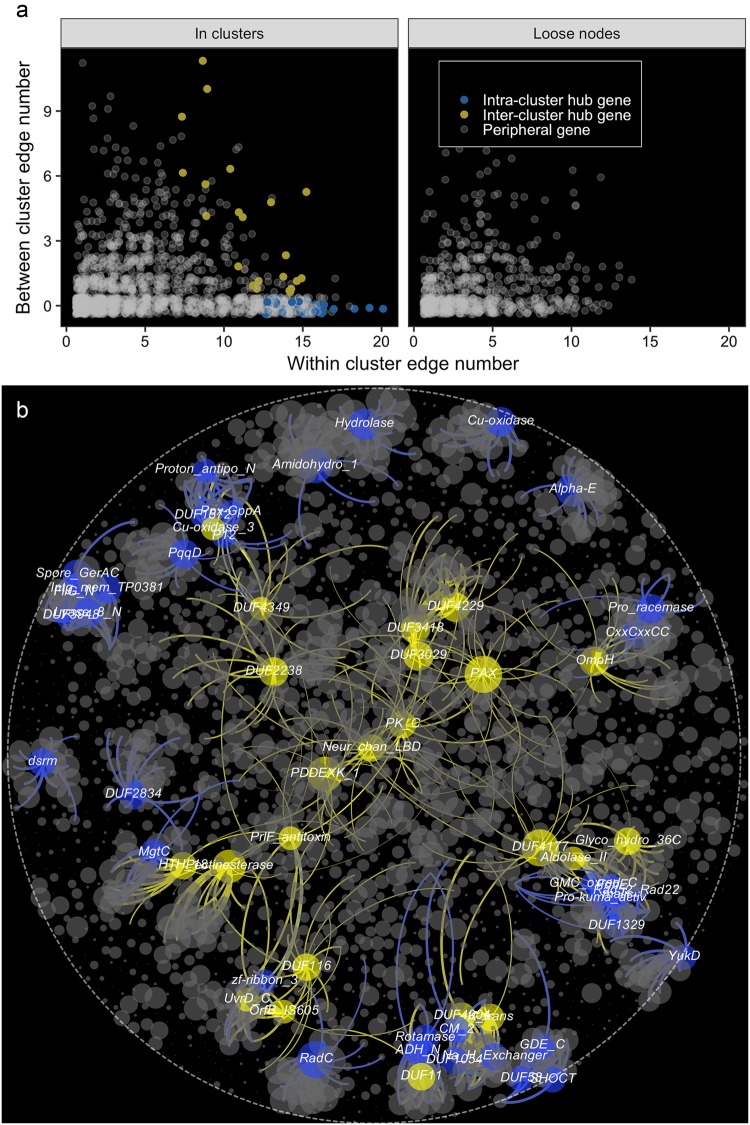
Highly connected hub genes in the genetic correlation network. **a** The within and between cluster connection numbers of nodes in the 27 dominant clusters and loose connected nodes. Hub genes wereare the network nodes that possessed the highest connection numbers in each of 27 dominant clusters. The hub genes were identified either as inter-cluster hub genes with connections between clusters (yellow) or intra-cluster hub genes without connections between clusters (blue). The point positions were adjusted by jittering to prevent overlap. **b** The connections of intra-cluster (blue) and inter-cluster (yellow) hub genes in the global genetic correlation network

To explore functional relationships between clusters, we detected the hierarchical structure of the clusters by tuning the resolution values (*R*) of the cluster detecting method from 1 to 15 (Fig. 4). The 27 clusters described above were detected by setting *R* as 1 (Fig. 4). At a relatively low-resolution level (*R* = 5), several sibling clusters collapsed into parent clusters with the same or closely associated functions, such as cluster 1 (iron-sulfur cluster binding) and 6 (catalytic activity), cluster 9 (nitrogen utilization process) and 10 (ion transport process), cluster 7 (translation) and 17 (development process), cluster 15 and 16 (phosphorus metabolic process), and cluster 26 and 27 (membrane parts). At resolution level *R* = 9, we found two larger parent clusters, in which the sibling clusters did not have closely associated functions, but were from the same cell compartments. For example, one of these two parent clusters combined with sibling clusters for membrane parts (cluster 14, 22, 26, and 27) and associated functions such as transport processes (cluster 15 and 16); but another parent cluster combined with sibling clusters for intracellular functions such as nucleic acid metabolic processes (cluster 4 and 21) and oxidation-reduction processes (cluster 7). At a high-resolution level (*R* = 13), those two large parent clusters were further combined into one parent cluster together with clusters for metal transport and binding processes (cluster 1 and 20). At this hierarchical level, the clusters for protein metabolic processes (cluster 2), xenobiotic degradation (cluster 18), and nitrogen utilization processes (cluster 9) were still independent from the large parent cluster. The cluster for nitrogen utilization processes was closely associated with the cluster enriched in ion channel proteins (cluster 10), catalytic activity (cluster 11), and oxidation-reduction (cluster 12).

### Negative correlation connections in the genetic correlation network

**Fig. 5:**
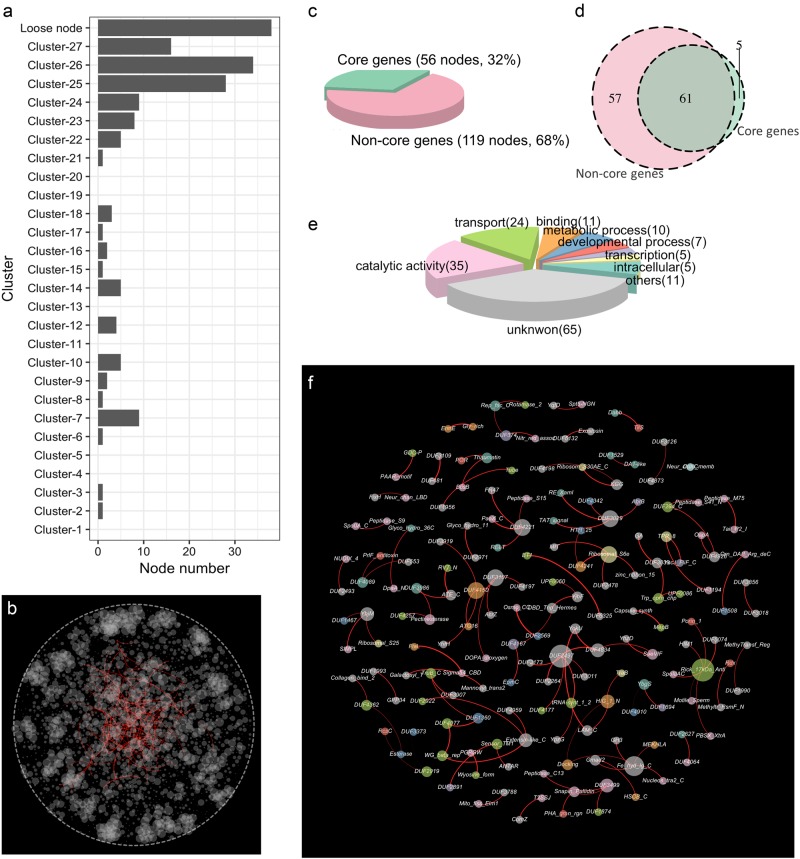
Negative correlation connections in the genetic correlation network. **a** Positions of negative correlation connections. **b** Distribution of negative correlation connections. **c** The abundances of core and non-core genes linked with negative correlation connections. **d** The abundance of negative correlation connections between non-core genes (n-n), between non-core and core genes (n-e), and between core genes (e-e). **e** The genetic functional classification of the genes linked with negative correlation connections. **f** The subnetwork of negative correlation network. The color of nodes shows the functional classification. The size of nodes shows the number of negative correlation connections

Negative correlation connections mainly linked genes in cluster 25–27 (Fig. 5a) and connected genes between different clusters (Fig. 5b). Most of the nodes with negative connections were non-core genes (Fig. 5c), half of the negative connections were between core and non-core genes, and only 5 negative connections were between core genes (Fig. 5d). Excluding 29 genes with unknown functions, the proteins encoded by the gene associating with negative connections were mainly functional for catalytic activities, metabolic processes, membrane proteins, developmental processes, and transport processes (Fig. 5e). The genes encoding the non-core genes, *Rick_17kDa_Anti*, *Fe_hyd_lg_C*, and *Ribosomal_S6e*, possessed significantly more negative connections than other genes (Wilcoxon rank sum test, *P* < 0.001) (Fig. 5f, Table S4). The genes linked by negative connections were generally from distinct functional classes (Fig. 5f).

### Predicting unknown gene functions

**Fig. 6:**
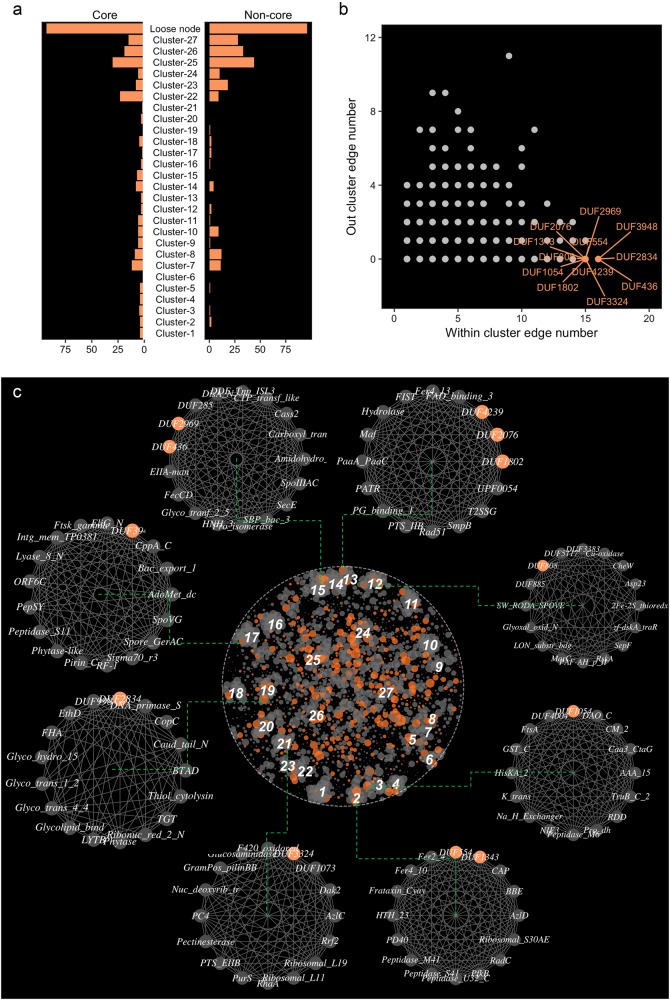
Predicting functions of genes encoding domain of unknown functions (*DUF*). **a** The distribution of *DUF*genes. **b** Identifying the intra-cluster *DUF* genes. **c** The position and neighbors of intra-cluster *DUF* genes in the global genetic correlation network

The global correlation network consisted of 573 DUF genes, most of which were loosely connected nodes or belonged to the articulating clusters (Fig. 6a). We identified 12 potentially function-specific DUF genes, which possessed large within cluster edge numbers but no between cluster edges (Fig. 6b). All of these genes were localized in intensively connected clusters (Fig. 6c). This association with clusters of known function allowed for reasonable predictions of the functional potential of the 12 DUF genes (Fig. 6c, Table S5). Seven of these predictions were successfully validated by structural homology modeling with SWISS-MODEL (Table S6). *DUF1343* and *DUF554* in cluster 2, localized in the vicinity of the peptidase genes (*Peptidase_S41, Peptidase_U32_c*, and *Peptidase M41)* and ferritin genes *(Fer2*4 and *Fer4_10*) potentially played roles in protein metabolism processes (Fig. 6c). Homology modeling results show that *DUF1343* contains domains with homology to orotate phosphoribosyl-transferase and precorrin-6A reductase, which play important roles in protein metabolism (Table S6). *DUF2969* and *DUF436* in cluster 15, were localized in the vicinity of transferase genes *Carboxyl_trans, CTP_transf_like*, and *Glyco_tranf_2_5*, which were potentially associated with transferase activity (Fig. 6c). *DUF2969* contained domains with homology to glucuronidase, threonylcarbamoyl-transferase, and ligand binding protein, which are all closely associated with transferase activity (Table S6). *DUF1802, DUF2076*, and *DUF4239* were in cluster 13 localized with the haloacid dehalogenase-like hydrolase gene *Hydrolase* and the phenylacetic acid catabolic protein gene *PaaA_PaaC*, with potential roles in the metabolism of organic substances (Fig. 6c). *DUF1802* contains domains with a homology to DNA ligase and endonuclease, which are involved in repairing DNA damage caused by phenylacetic acid (Table S6). *DUF2076* contains a domain with homology to a microphthalmia associated transcription factor, which regulates metabolism processes in mitochondria (Table S6). *DUF4329* contains a domain with homology to bestrophin, a chloride channel protein, and hence is associated with haloacid dehalogenation (Table S6). *DUF2834* in cluster 19 has potential functions in glycosyl compound metabolism, since it is closely connected with genes for glycosyl hydrolase (*Glyco_hydro_15*), transferase (*Glyco_trans_1_2* and *Glyco_trans_4_4*), and binding (*Glycolipid_bind*). *DUF2834* contains domains with homology to membrane protein arginine antiporters and epidermal growth factor receptors (Table S6). Given that glycosyl compounds are essential components of cell membranes, membrane protein is expected to be closely associated with glycosyl compound metabolism. *DUF3324* in cluster 21 is localized in the vicinity of the ribosomal protein genes *Ribosomal_L11* and *Ribosomal_L19*, the transcriptional regulator genes *Rrf2* and *PC4*, suggesting a potential role of *DUF3324* in the transcription process. *DUF3324* contains a domain with homology to a chaperone that has been reported to regulate transcription factor RUNX1 (Table S6). *DUF1504* in cluster 3, localized in the vicinity of oxidase genes *Caa2_CtaG* and *DAO_C*, dehydrogenase gene *Pro_dh*, and transferase gene *GST_C*, were potentially involved in the oxidation-reduction process. *DUF808*, in cluster 12, was closely connected with oxidase gene Cu-oxidase and Glyoxal_oxid_N and ferredoxin gene 2Fe-2S_thioredx, were potentially associated with oxidation-reduction processes. *DUF3948* in cluster 17 was closely connected with genes for the reproduction process, such as the *SpoVG* gene for sporulation and the *Spore_GerAC* gene for spore germination, which play potential roles in development.

## Discussion

We analyzed the connectivity pattern in a genetic correlation network based on the 45 forest soil metagenomes and identified 27 clusters of enriched genes associated with various functions. Compartmentalizing clusters at different resolutions revealed a hierarchical structure of cluster organization. Cluster hubs could reflect both functionality and topological features of corresponding clusters. Negative correlation connections mainly wired genes from articulating clusters. Moreover, the genetic correlation network can be used to predict previously unknown genetic functions from the functions of adjacent genes.

The clusters in our genetic correlation network formed a hierarchic structure as observed in genetic interaction networks from yeast to human [16, 47, 48]. Consistent with the function hierarchy of the genetic interaction network in yeast cells [16], parent clusters were enriched with sibling clusters with closely associated functions at low modularity resolutions, but enriched with sets of sibling clusters from the same subcellular compartments at greater resolutions. This finding suggests that the properties of genetic interactions at the cellular scale could be extrapolated to the community scale. The hierarchical structure of network cluster associations provides an insight into the relationships among functional clusters in the metagenomes. The genetic interactions occurring between clusters are conserved at a lower level than the interactions within clusters [49]. This suggests that the selective pressure for maintaining interactions within a single cluster is much greater than between clusters [49]. The connections between clusters found in the present study might represent evolutionary conserved genetic interactions between clusters and could be essential for deciphering functional organization in soil metagenomes.

An important property associated with the genetic correlation network is the intensely connected clusters in the network. Distinct topological features suggest different functionality between densely connected clusters dominated by core genes and articulating clusters dominated by non-core genes in the genetic correlation network. A previous study showed that the topological features of the core gene subnetwork are distinct from those of the non-core gene subnetwork in yeast cells [16]. Less between cluster connections for densely connected clusters suggest that some clusters contain genes enriched in particular conditions. Despite the high diversity of taxonomic groups and functional profiles for various microorganisms in soils, the conservation of genes for fundamental biological processes has been observed across different taxa [50]. Accordingly, those conserved core genes associating with a specific function, such as nucleic acid and energy metabolic processes, are expected to be intensely connected within the clusters, with a high degree of functional independence [47]. Conversely, the articulating clusters interacting with a large number of core clusters, such as membrane transport and secretion, confirm that these processes are important for mediating cross-cluster connections [51]. The shorter diameter of the global network, when compared with the core gene subnetwork, also suggests that non-core genes generally complement the connection among clusters. Although non-core genes are not necessarily essential for cellular function, they could enhance the flexibility and efficiency of networks by providing functional pathway redundancy [52]. Since the essentiality of a gene is environment-dependent [53], non-core genes may also include genes which are essential in particular environments. Whereas many microbial functions and their associated genetic interactions are still unclear, the clusters identified from the genetic correlation network provide an alternative approach for exploring genes potentially involved in corresponding soil microbial community functions. However, the links between genetic correlation network inference from gene co-occurrence do not necessarily represent genetic interactions or regulations due to false positives and indirect connections [54]. Although we determined cut-off thresholds using the RMT method and reduced indirect connections with a deconvolution method, the links determined in the genetic correlation network still need to be treated with caution.

The functions of clusters could be also validated with the cluster hub genes, which have been proposed to be keystone nodes due to their important roles in network topology [25]. The hubs in intra-cluster clusters were mainly wired with genes within clusters, representing the intra-cluster feature of these clusters. Hubs in articulating clusters were mainly identified as inter-cluster nodes, representing the mediation functions of these clusters. These inter-cluster nodes, wired different functional clusters, are therefore essential for understanding the functional organization in the genetic correlation network. Hub genes with more connections would be less exposed to the mutations associated with adaptive evolution than peripheral genes with less connections in the genetic interaction network. Accordingly, the hubs could provide an overview of the network and could indicate the potential functions of the corresponding clusters.

It is notable that the environmental factors closely associated with the genes in genetic correlation networks, including the available K, C/N ratio, and dissolved Fe and Al, were different from the environmental drivers for the soil microbial community reported in previous studies, such as temperature [55] and soil pH [56]. This suggests that the underlying mechanism for genetic interaction patterns is distinct from that for microbial community assembly. Iron plays important roles in a wide range of gene regulatory processes [57]. The dissolved Fe concentration is also important for the variation in topology of microbial co-occurrence networks [27]. Metabolite analyses have revealed that K^+^ deficiency affects the metabolic state of bacterial cells by impairing oxidative phosphorylation [58]. The C/N ratio of the cell is also essential for regulating metabolism in microbial cells [59]. The associations for total and available nitrogen were all linked with genes in the nitrogen processes cluster, suggesting that links predicted between environmental factors and genes are meaningful.

Positive connections in the genetic correlation network generally indicate functional sharing and association, while negative connections generally reflect regulatory and suppression interactions [60]. The genes wired by negative connections were mainly non-core genes in articulating clusters, and were generally from different functional classes and network clusters. Therefore, we speculate that negative connections were potentially regulatory interactions between functional clusters rather than suppression interactions, which generally appears between genes with functional redundancy. For instance, *Rick_17kDa_Anti* gene encoding an antigen protein [61]; *Fe_hyd_lg_C* gene encoding a ferredoxin catalyzes a range of redox reactions [62]; *Ribosomal_S6* encoding ribosomal protein S6 is involved in regulating translation [63]. Although only small numbers were observed, negative connections could potentially be useful in manipulating soil microbial community functions by controlling those interactions.

A large number of genes with unknown functions exist in global databases [8]. Similar to networks from yeast [16], the genetic correlation network can predict the unknown functions of genes, when these genes display highly intra-cluster connection features. The connection density of the genetic correlation network was much lower than in the genetic interaction network of yeast cells [16]. This could be interpreted as suggesting that evolutionary conserved genetic interactions across a wide range of species are present in the genetic correlation network. Accordingly, the functional predictions made in this study could be valuable for gene function annotation regardless of phylogenetic distance. However, these predictions were more accurate when the DUF genes were located in core gene clusters and had only intra-cluster edges. Moreover, the network focusing on the core genes better captures the high functional redundancy of the microbial community.

In summary, the genetic correlation network in the present study provides insight into the functional organization of forest soil communities. Coherent sets of positive and negative genetic correlation connections wired both within and between these clusters revealed a hierarchical structure similar to the organization of the genetic network at cellular scale. This finding suggests that the functions of microbial communities could be organized based on the regulations at cellular scale, which has been extensively investigated with systems biology. Distinct topological features of intensively connected clusters and articulating clusters indicated different functional associations. Cluster hub genes that manifested the functions and the wiring features of corresponding clusters could be employed as indicators for a network skeleton. We also presented a novel approach for predicting genes and domains of unknown function in metagenomes. We anticipate that the connection pattern of the genetic correlation network could elucidate the functional organization for soil metagenomes and may be exploited to systematically predict microbial community functions.

## Electronic supplementary material

Figure S1-S8, Table S1-S6
