# Supplementary material for: Genetic correlation network prediction of forest soil microbial functional organization
Source: ISME J. 2018 Jul 25;12(10):2492–505. doi: 10.1038/s41396-018-0232-8 (PMC6155114; doi:10.1038/s41396-018-0232-8)
Supplement: Supplementary file 1 — Figure S1-S8, Table S1-S6 [file 41396_2018_232_MOESM1_ESM.docx]

# *-Supplementary Materials-*

# Genetic correlation network prediction of forest soil microbial functional organization

Bin Ma ^a, b^, Kankan Zhao ^a, b^, Xiaofei Lv ^a, b^, Weiqin Su ^a, b^, Zhongmin Dai ^a, b^, Jack A Gilbert ^c, d^, Philip C. Brookes ^a, b^, Karoline Faust ^e^, Jianming Xu ^a, b *^

^a^ Institute of Soil and Water Resources and Environmental Science, College of Environmental and Resource Sciences, Zhejiang University, Hangzhou 310058, China

^b^ Zhejiang Provincial Key Laboratory of Agricultural Resources and Environment, Hangzhou 310058, China

^c^ The Microbiome Center, Department of Surgery, University of Chicago, IL 60637, USA

^d^ Bioscience Division, Argonne National Laboratory, Lemont, IL, 60439, USA

^e^ Department of Microbiology and Immunology, Rega Institute, KU Leuven, Campus Gasthuisberg, Leuven, Belgium

*Corresponding author: Jianming Xu, Email: [jmxu@zju.edu.cn](mailto:jmxu@zju.edu.cn)

***Table of Contents***

*Supplementary Figures*

- Figure S1. The position of 45 sampling sites across eastern China.
- Figure S2. The degree of distribution of genetic network and random network with the same nodes and links.
- Figure S3. The position of Core (orange) and non-Core (green) hub nodes in the genetic network.
- Figure S4. The taxonomic profiles of gene clusters in the genetic correlation network.
- Figure S5. Summary of genera in the clusters of the genetic correlation network.
- Figure S6. The principal coordination analysis and influential environmental factors for the composition of genera and genes in forest soil samples from five vegetation types.
- Figure S7. The bipartite network between environmental variables and the significantly correlated genes in metagenomics.
- Figure S8. Wiring diagram of global metabolic pathways in soil metagenomics. Green lines indicate the occurrence of corresponding metabolic pathways.

*Supplementary Tables*

- Table S1. Topological properties of the entire genetic network, Core subnetwork, non-Core subnetwork, and random network.
- Table S2. The functions of cluster hub nodes.
- Table S3. The environmentally influenced genes that are involved in the genetic correlation network.
- Table S4. The neighbour nodes of functional specific DUF genes.
- Table S5. The homological domains of DUF genes.
- Table S6. Summary of sequencing data


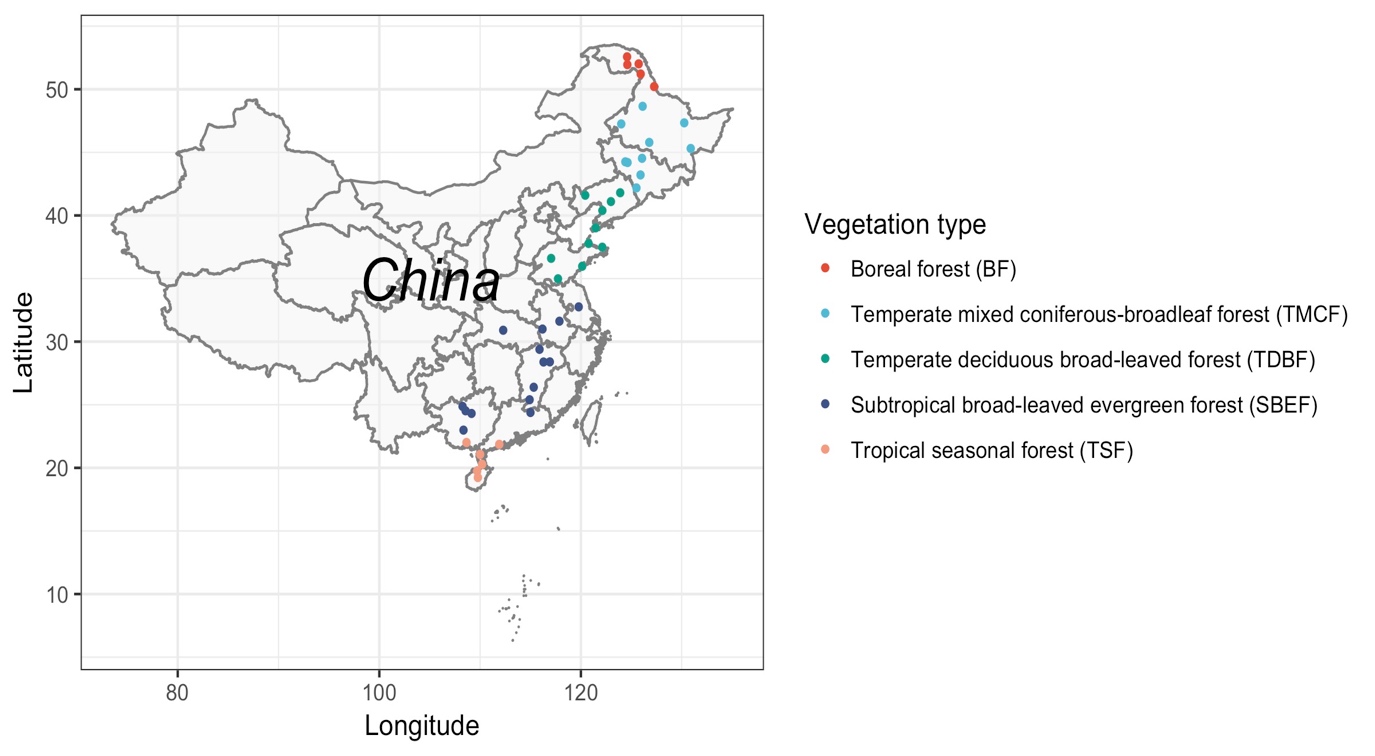


Figure S1. The position of 45 sampling sites across eastern China.


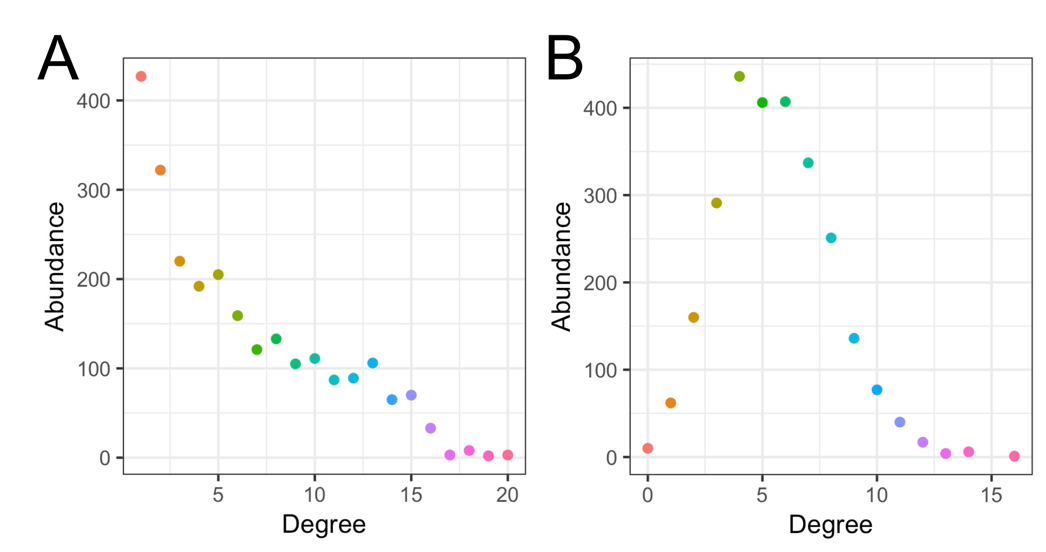


Figure S2. The degree distribution of (A) genetic network and (B) Erdős-Renyi network with same nodes and links.


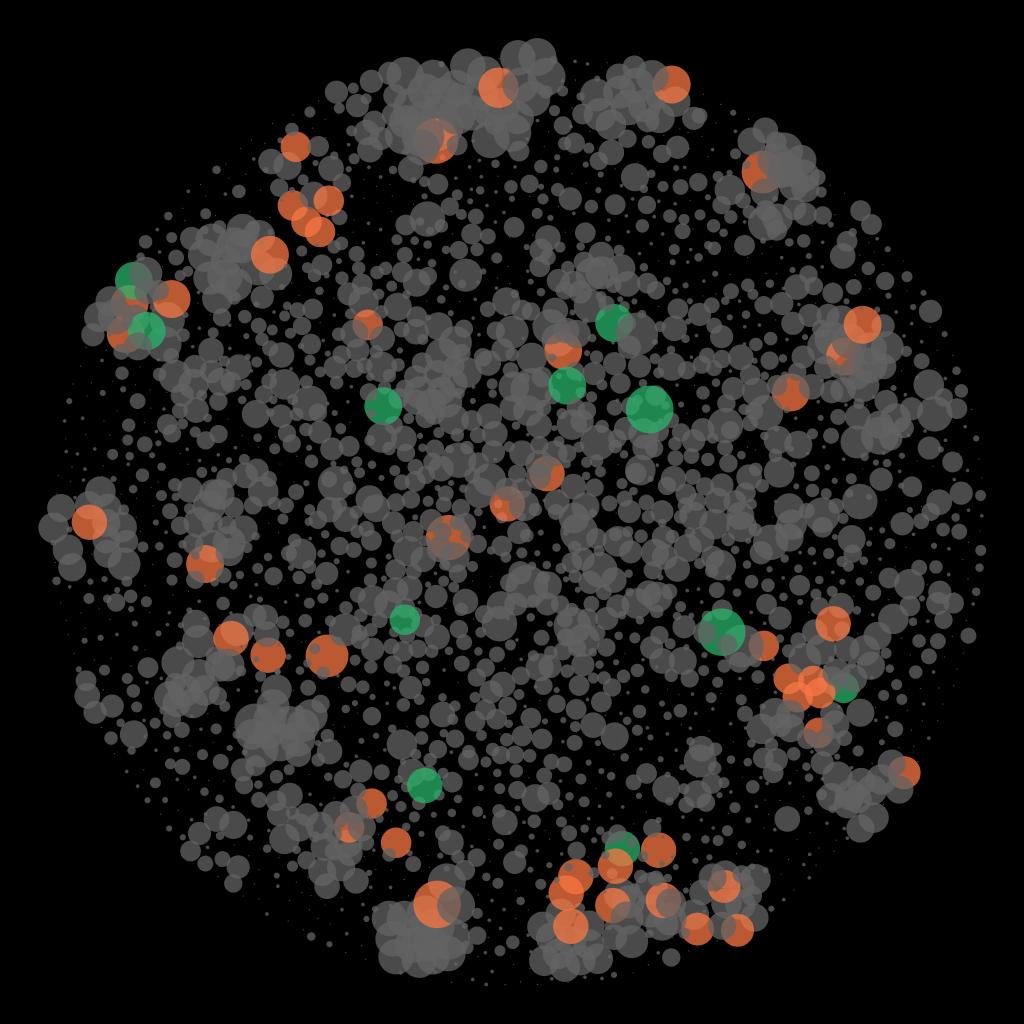


Figure S3. The position of Core (orange) and non-Core (green) hub nodes in the genetic network.


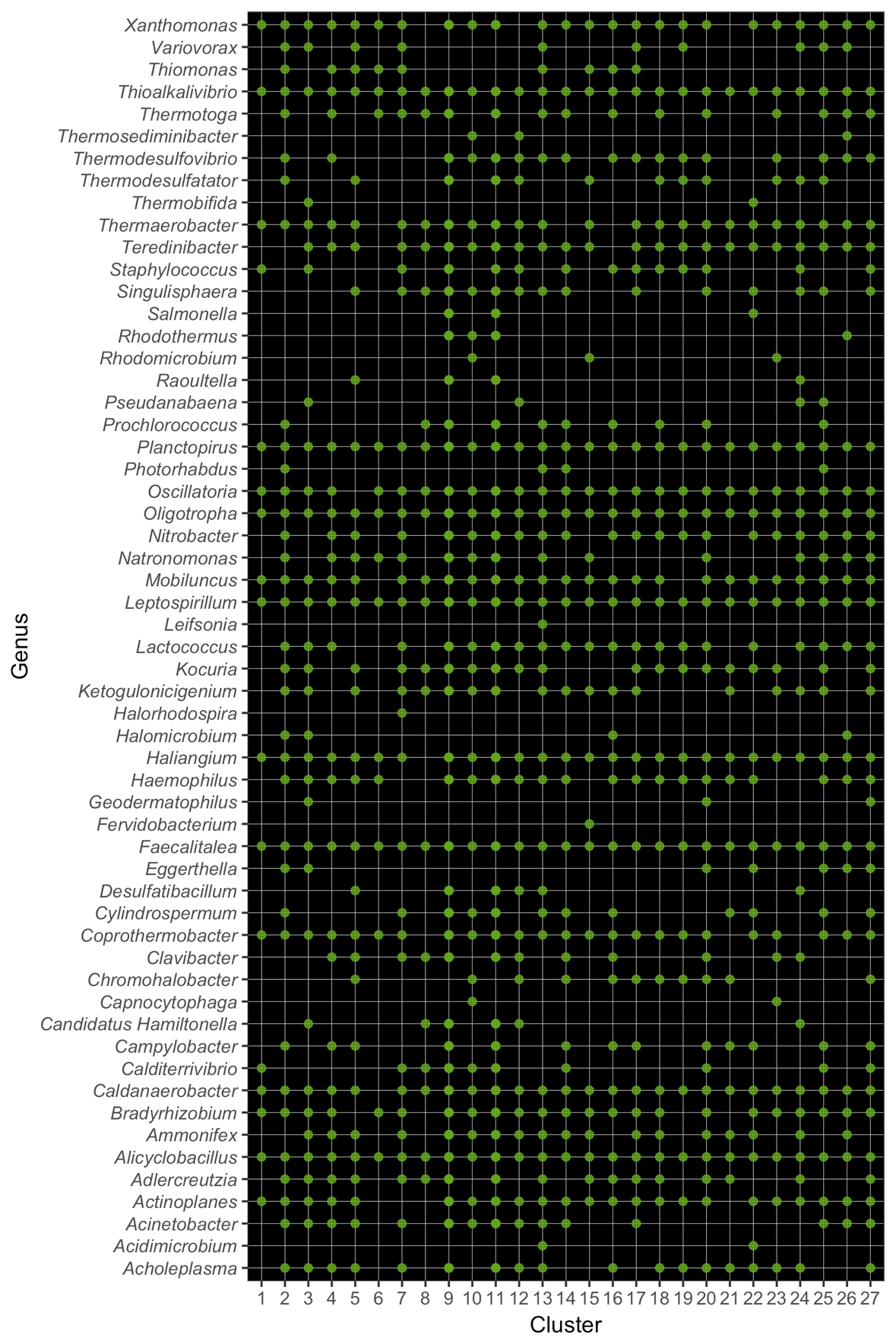


Figure S4. The taxonomic profiles of gene clusters in the genetic correlation network.


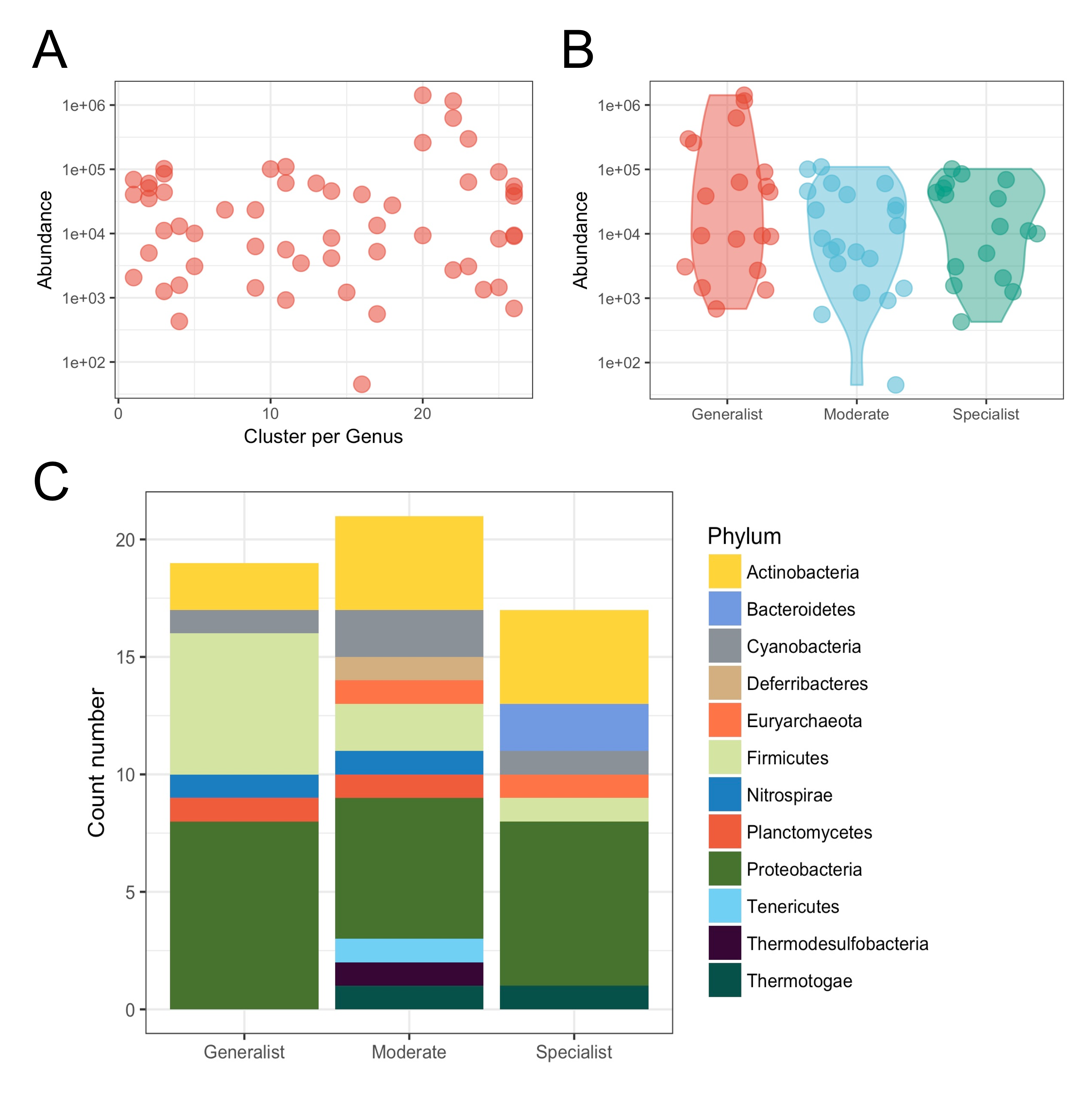


Figure S5. Summary of genera in the clusters of the genetic correlation network. (A) The relationship between the abundance and the number of presenting cluster of genus. (B) The abundance distribution of genera classified as functional generalist (cluster per genus ≥ 20), moderate (6 ≥ cluster per genus ≥ 19), and specialist (cluster per genus ≤ 5). The phyla profiles for genera classified as functional generalist, moderate, and specialist.


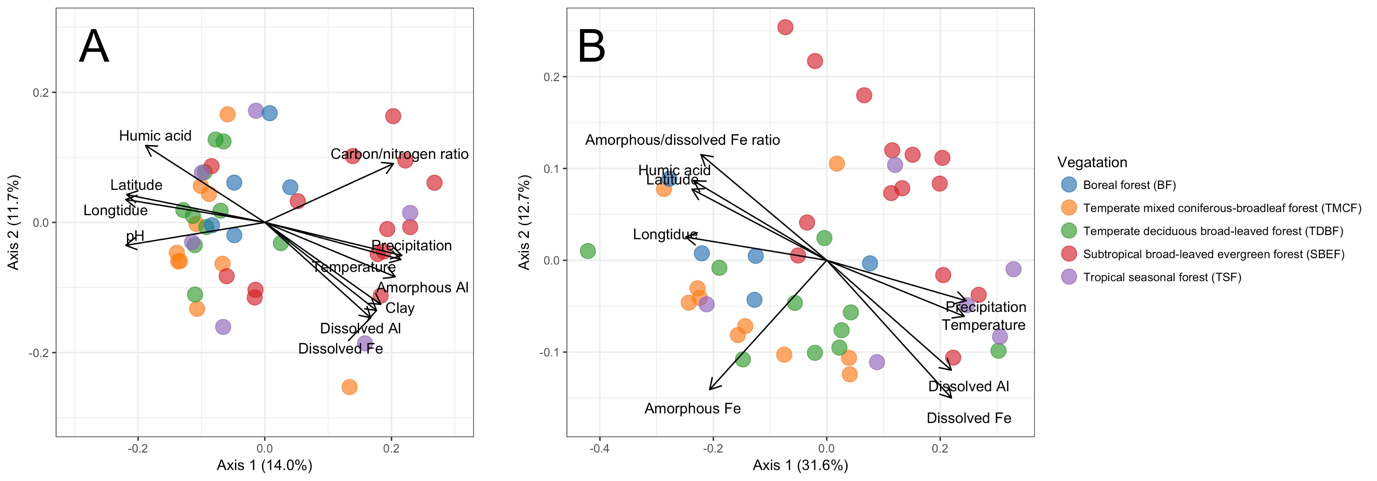


Figure S6. The principal coordination analysis and influential environmental factors for the composition of (A) genera and (B) genes in forest soil samples from five vegetation types.


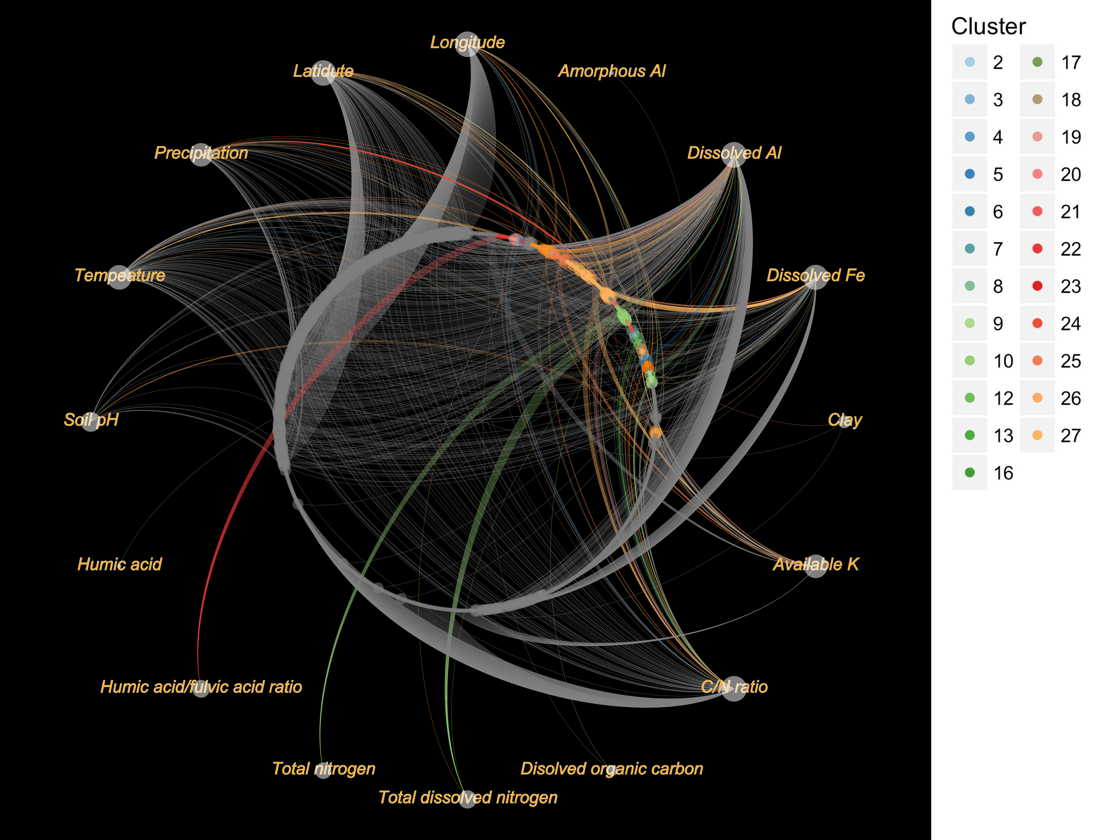


Figure S7. The bipartite network between environmental variables and the significantly correlated genes in metagenomics. The central nodes indicate the environmental variables. The nodes in the outer circle indicate the significantly correlated genes in metagenomics. The grey nodes in the outer circle are the genes that have not been involved in the genetic network. The nodes in the outer circle with other colors indicate the genes belonging to different clusters. The colors of lines represent the color of linked genes.


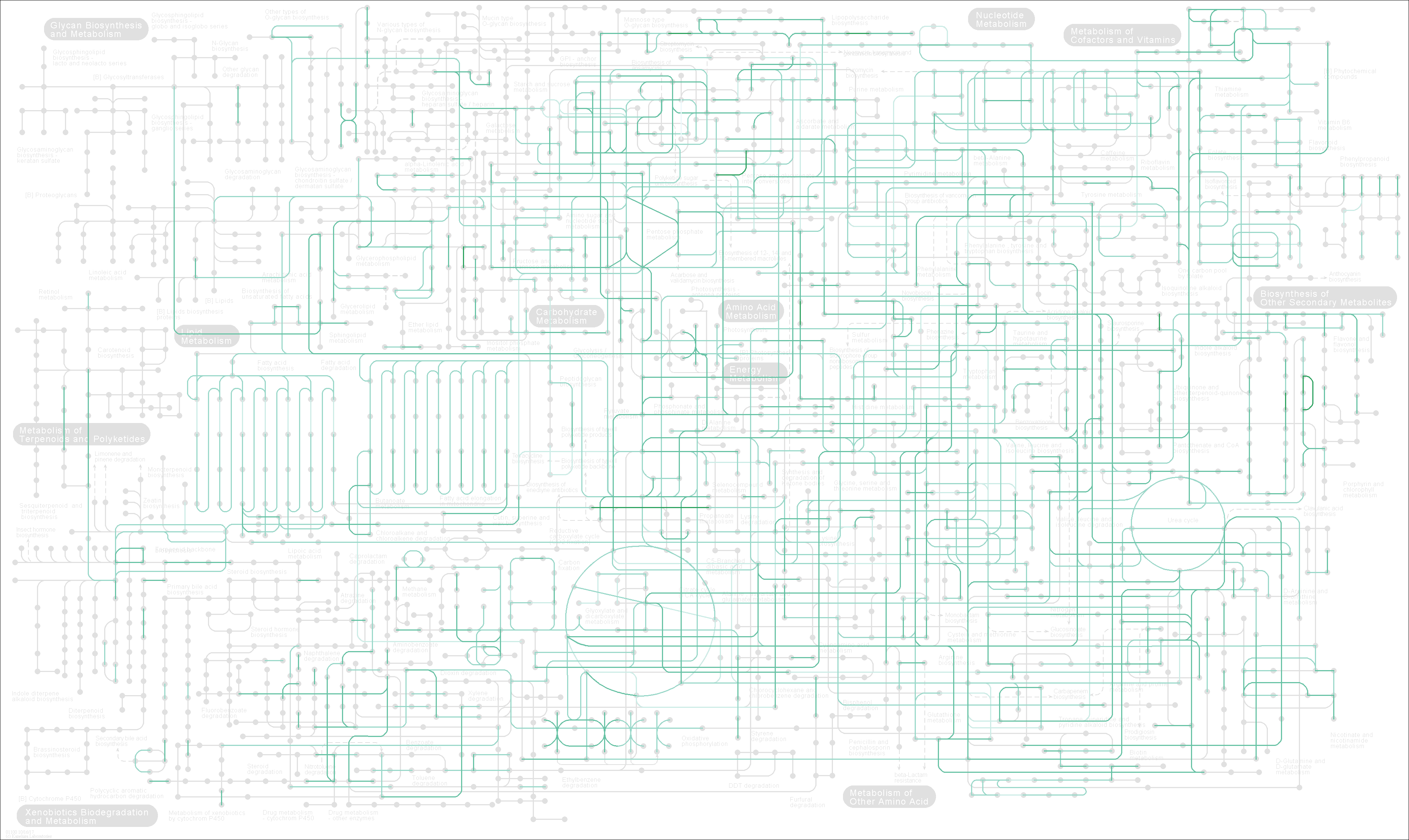


Figure S8. Wiring diagram of global metabolic pathways in soil metagenomics. Green lines indicate the occurrence of corresponding metabolic pathways. The intensity of green indicates the numbers of reads assigned to corresponding genes of metabolic processes.

Table S1. Topological properties of Erdős-Renyi network, entire genetic network, and subnetwork for Core and non-Core genes.

| **Topological Parameters** | **Erdos-Renyi network** | **Entire Genetic Network** | **Subnetwork of Core Gene** | **Subnetwork of Non-Core Gene** |
| --- | --- | --- | --- | --- |
| Node number | 2641 | 2641 | 1635 | 826 |
| Edge number | 7314 | 7314 | 4135 | 1374 |
| Average degree | 5.5 | 5.9 | 5.1 | 3.3 |
| Average Weight. Degree | 4.3 | 4.6 | 4.2 | 2.2 |
| Diameter | 10 | 16 | 25 | 15 |
| Graph Density | 0.002 | 0.004 | 0.003 | 0.004 |
| Modularity | 0.39 | 0.87 | 0.93 | 0.81 |
| Clustering Coefficient | 0.003 | 0.41 | 0.49 | 0.28 |
| Average Path Length | 4.8 | 6.2 | 8.8 | 6.2 |

Table S2. The function annotation for the cluster hub nodes.

| **Cluster**  **Number** | **Cluster Functions** | **Functional Type** | **Gene Type** | **Pfam ID** | **Pfam**  **Access**  **Number** | **Description of genes** | **GO Class** |
| --- | --- | --- | --- | --- | --- | --- | --- |
| **1** | Metal cluster binding | Functional specific gene | Core | *GDE_C* | PF06202.12 | Amylo-alpha-1,6-glucosidase | Glycogen metabolic process |
| **1** | Metal cluster binding | Functional specific gene | Core | *SHOCT* | PF09851.7 | Short C-terminal domain | Protein binding |
| **1** | Metal cluster binding | Functional specific gene | Core | *DUF58* | PF01882.16 | Protein of unknown function DUF58 | Unknown |
| **2** | Protein metabolic process | Functional specific gene | Core | *CM_2* | PF01817.19 | Chorismate mutase type II | Catalytic activity |
| **2** | Protein metabolic process | Functional specific gene | Core | *DUF1054* | PF06335.10 | Protein of unknown function (DUF1054) | Unknown |
| **2** | Protein metabolic process | Pleiotropic gene | Core | *K_trans* | PF02705.14 | K+ potassium transporter | Transmembrane transport |
| **2** | Protein metabolic process | Functional specific gene | Core | *Na_H_Exchanger* | PF00999.19 | Sodium/hydrogen exchanger family | Metal ion transport |
| **2** | Protein metabolic process | Pleiotropic gene | Non-core | *DUF4004* | PF13171.4 | Protein of unknown function (DUF4004) | Unknown |
| **3** | Oxidation-reduction process | Functional specific gene | Core | *RadC* | PF04002.13 | RadC-like JAB domain | Hydrolase activity |
| **4** | Nucleic acid metabolic process | Pleiotropic gene | Core | *DUF11* | PF01345.16 | Domain of unknown function DUF11 | Unknown |
| **4** | Nucleic acid metabolic process | Functional specific gene | Core | *Rotamase* | PF00639.19 | PPIC-type PPIASE domain | Protein metabolic |
| **4** | Nucleic acid metabolic process | Functional specific gene | Core | *ADH_N* | PF08240.10 | Alcohol dehydrogenase GroES-like domain | Oxidoreductase activity |
| **5** | Catalytic activity | Functional specific gene | Core | *YukD* | PF08817.8 | WXG100 protein secretion system (Wss), protein YukD | Transferase activity |
| **6** | Catalytic activity | Pleiotropic gene | Core | *Aldolase_II* | PF00596.19 | Class II Aldolase and Adducin N-terminal domain | Lyase activity |
| **6** | Catalytic activity | Functional specific gene | Core | *DUF1329* | PF07044.9 | Protein of unknown function (DUF1329) | Unknown |
| **6** | Catalytic activity | Functional specific gene | Core | *FdhE* | PF04216.10 | Protein involved in formate dehydrogenase formation | Oxidoreductase activity |
| **6** | Catalytic activity | Functional specific gene | Core | *Pro-kuma_activ* | PF09286.9 | Pro-kumamolisin, activation domain | Hydrolase activity |
| **6** | Catalytic activity | Functional specific gene | Non-core | *Rad52_Rad22* | PF04098.13 | Rad52/22 family double-strand break repair protein | DNA repair complex |
| **6** | Catalytic activity | Functional specific gene | Core | *GMC_oxred_C* | PF05199.11 | GMC oxidoreductase | Oxidoreductase activity |
| **6** | Catalytic activity | Functional specific gene | Core | *malic* | PF00390.17 | Malic enzyme, N-terminal domain | Oxidoreductase activity |
| **7** | Translation process | Pleiotropic gene | Core | *Glyco_hydro_36C* | PF16874.3 | Glycosyl hydrolase family 36 C-terminal domain | Hydrolase activity |
| **7** | Translation process | Pleiotropic gene | Core | *PK_C* | PF02887.14 | Pyruvate kinase, alpha/beta domain | Transferase activity |
| **7** | Translation process | Pleiotropic gene | Core | *Neur_chan_LBD* | PF02931.21 | Neurotransmitter-gated ion-channel ligand binding domain | Ion transport |
| **8** | Transport process | Pleiotropic gene | Non-core | *DUF4177* | PF13783.4 | Domain of unknown function (DUF4177) | Unknown |
| **9** | Nitrogen utilization process | Functional specific gene | Core | *Alpha-E* | PF04168.10 | A predicted alpha-helical domain with a conserved ER motif. | Protein binding |
| **10** | Transport process | Functional specific gene | Core | *CxxCxxCC* | PF03692.13 | Putative zinc- or iron-chelating domain | Protein binding |
| **10** | Transport process | Pleiotropic gene | Core | *OmpH* | PF03938.12 | Outer membrane protein (OmpH-like) | Membrane organization |
| **10** | Transport process | Functional specific gene | Core | *Pro_racemase* | PF05544.9 | Proline racemase | Isomerase activity |
| **12** | Oxidation-reduciotn process | Functional specific gene | Core | *Cu-oxidase* | PF00394.20 | Multicopper oxidase | Oxidoreductase activity |
| **13** | Oxidation-reduciotn process | Functional specific gene | Core | *Hydrolase* | PF00702.24 | haloacid dehalogenase-like hydrolase | Hydrolase activity |
| **14** | Transport process | Functional specific gene | Core | *Amidohydro_1* | PF01979.18 | Amidohydrolase family | Hydrolase activity |
| **15** | Phosphorus metabolic process | Functional specific gene | Core | *DUF1572* | PF07609.9 | Protein of unknown function (DUF1572) | Unknown |
| **15** | Phosphorus metabolic process | Functional specific gene | Core | *P12* | PF12669.5 | Virus attachment protein p12 family | Membrane |
| **15** | Phosphorus metabolic process | Functional specific gene | Core | *Ppx-GppA* | PF02541.14 | Ppx/GppA phosphatase family | Protein binding |
| **15** | Phosphorus metabolic process | Functional specific gene | Core | *Proton_antipo_N* | PF00662.18 | NADH-Ubiquinone oxidoreductase (complex I), chain 5 N-terminus | Oxidoreductase activity |
| **15** | Phosphorus metabolic process | Pleiotropic gene | Core | *Cu-oxidase_3* | PF07732.13 | Multicopper oxidase | Oxidoreductase activity |
| **15** | Phosphorus metabolic process | Pleiotropic gene | Core | *DUF4349* | PF14257.4 | Domain of unknown function (DUF4349) | Unknown |
| **16** | Phosphorus metabolic process | Functional specific gene | Core | *PqqD* | PF05402.10 | Coenzyme PQQ synthesis protein D (PqqD) | Biosynthetic process |
| **17** | Development process | Functional specific gene | Core | *FliG_N* | PF14842.4 | FliG N-terminal domain | Motor activity |
| **17** | Development process | Functional specific gene | Core | *Intg_mem_TP0381* | PF09529.8 | Integral membrane protein (intg_mem_TP0381) | Membrane organization |
| **17** | Development process | Functional specific gene | Non-core | *Lyase_8_N* | PF08124.9 | Polysaccharide lyase family 8, N terminal alpha-helical domain | Lyase activity |
| **17** | Development process | Functional specific gene | Non-core | *Spore_GerAC* | PF05504.9 | Spore germination B3/ GerAC like, C-terminal | Spore germination |
| **17** | Development process | Functional specific gene | Core | *DUF3948* | PF13134.4 | Protein of unknown function (DUF3948) | Unknown |
| **18** | Catalytic activity | Functional specific gene | Core | *DUF2834* | PF11196.6 | Protein of unknown function (DUF2834) | Unknown |
| **19** | Immunity process | Functional specific gene | Core | *dsrm* | PF00035.24 | Double-stranded RNA binding motif | RNA binding |
| **20** | Transport process | Functional specific gene | Core | *MgtC* | PF02308.14 | MgtC family | Membrane |
| **20** | Transport process | Pleiotropic gene | Core | *HTH_18* | PF12833.5 | Helix-turn-helix domain | DNA binding |
| **21** | Nucleic acid metabolic process | Pleiotropic gene | Core | *Pectinesterase* | PF01095.17 | Pectinesterase | Hydrolase activity |
| **22** | Intracellular part | Pleiotropic gene | Non-core | *PrlF_antitoxin* | PF15937.3 | prlF antitoxin for toxin YhaV_toxin | Protein binding |
| **22** | Intracellular part | Pleiotropic gene | Core | *UvrD_C* | PF13361.4 | UvrD-like helicase C-terminal domain | Hydrolase activity |
| **22** | Intracellular part | Functional specific gene | Core | *zf-ribbon_3* | PF13248.4 | zinc-ribbon domain | Membrane |
| **22** | Intracellular part | Pleiotropic gene | Core | *OrfB_IS605* | PF01385.17 | Probable transposase | Transposition |
| **23** | Nucleic acid metabolic process | Pleiotropic gene | Non-core | *DUF116* | PF01976.15 | Protein of unknown function DUF116 | Unknown |
| **24** | Membrane part | Pleiotropic gene | Non-core | *PAX* | PF00292.16 | 'Paired box' domain | Protein binding |
| **25** | Stress response process | Pleiotropic gene | Core | *PDDEXK_1* | PF12705.5 | PD-(D/E)XK nuclease superfamily | Hydrolase activity |
| **26** | Immunity process | Pleiotropic gene | Non-core | *DUF2238* | PF09997.7 | Predicted membrane protein (DUF2238) | Unknown |
| **27** | Membrane part | Pleiotropic gene | Non-core | *DUF3029* | PF11230.6 | Protein of unknown function (DUF3029) | Unknown |
| **27** | Membrane part | Pleiotropic gene | Core | *DUF3418* | PF11898.6 | Domain of unknown function (DUF3418) | Unknown |
| **27** | Membrane part | Pleiotropic gene | Non-core | *DUF4229* | PF14012.4 | Protein of unknown function (DUF4229) | Unknown |

|  | | |
| --- | --- | --- |
| Table S3. The environmentally influenced genes that are involved in the genetic correlation network. | | |
| Environmental factors | Gene | Cluster |
| Longitude | APG17 | C22 |
| Longitude | ArAE_1 | C15 |
| Longitude | BetaGal_dom2 | C23 |
| Longitude | Curlin_rpt | C44 |
| Longitude | DUF11 | C43 |
| Longitude | DUF2339 | C14 |
| Longitude | DUF3142 | C23 |
| Longitude | DUF378 | C18 |
| Longitude | DUF3792 | C47 |
| Longitude | DUF3935 | C47 |
| Longitude | DUF459 | C43 |
| Longitude | Endonuclea_NS_2 | C33 |
| Longitude | Eno-Rase_NADH_b | C9 |
| Longitude | Exonuc_V_gamma | C15 |
| Longitude | FliM | C17 |
| Longitude | FliMN_C | C17 |
| Longitude | FtsL | C23 |
| Longitude | Fumble | C50 |
| Longitude | GerPC | C50 |
| Longitude | Glyco_hydro_42 | C6 |
| Longitude | HTH_5 | C32 |
| Longitude | Peptidase_S8 | C23 |
| Longitude | PUD | C40 |
| Longitude | PvlArgDC | C44 |
| Longitude | S-AdoMet_synt_N | C22 |
| Longitude | SatD | C17 |
| Longitude | SIR2 | C18 |
| Longitude | T6SS_TssG | C33 |
| Longitude | TelA | C23 |
| Longitude | TerB | C17 |
| Longitude | tRNA_NucTran2_2 | C37 |
| Longitude | YtxH | C18 |
| Longitude | zf-CHCC | C44 |
| Latidute | BMFP | C35 |
| Latidute | CbiG_C | C23 |
| Latidute | DUF2478 | C23 |
| Latidute | FlgT_C | C6 |
| Latidute | MbeB_N | C9 |
| Latidute | OST-HTH | C6 |
| Latidute | Ribonuc_red_lgN | C22 |
| Latidute | SBF | C52 |
| Latidute | Urocanase_N | C47 |
| Latidute | YgbA_NO | C17 |
| Precipitation | Chlam_OMP6 | C44 |
| Precipitation | Competence | C31 |
| Precipitation | DNA_pol3_beta | C31 |
| Precipitation | DUF3324 | C31 |
| Precipitation | ECF-ribofla_trS | C31 |
| Precipitation | F420_oxidored | C31 |
| Precipitation | Nuc_deoxyrib_tr | C31 |
| Precipitation | NUDIX | C31 |
| Precipitation | PTS_EIIB | C31 |
| Precipitation | PurS | C31 |
| Precipitation | RhaA | C31 |
| Soil pH | Band_7_C | C6 |
| Soil pH | Fe_hyd_lg_C | C35 |
| Soil pH | His_Phos_2 | C33 |
| Soil pH | YlaH | C6 |
| Humic acid | GvpG | C42 |
| Humic acid/fulvic acid ratio | Ald_Xan_dh_C | C10 |
| Humic acid/fulvic acid ratio | ATPase_gene1 | C10 |
| Humic acid/fulvic acid ratio | ChW | C10 |
| Humic acid/fulvic acid ratio | dsrm | C10 |
| Humic acid/fulvic acid ratio | DUF1071 | C10 |
| Humic acid/fulvic acid ratio | Epimerase | C10 |
| Humic acid/fulvic acid ratio | FMO-like | C10 |
| Humic acid/fulvic acid ratio | Hpr_kinase_C | C10 |
| Humic acid/fulvic acid ratio | LytR_cpsA_psr | C10 |
| Humic acid/fulvic acid ratio | MutL_C | C10 |
| Humic acid/fulvic acid ratio | Phage_Mu_F | C10 |
| Humic acid/fulvic acid ratio | Ribosomal_L36 | C10 |
| Humic acid/fulvic acid ratio | Sod_Cu | C10 |
| Total nitrogen | ATPgrasp_Ter | C30 |
| Total nitrogen | CitMHS | C30 |
| Total nitrogen | Cons_hypoth95 | C30 |
| Total nitrogen | DUF1932 | C30 |
| Total nitrogen | FA_hydroxylase | C30 |
| Total nitrogen | FGGY_C | C30 |
| Total nitrogen | MR_MLE_C | C30 |
| Total nitrogen | Oxidored_molyb | C30 |
| Total nitrogen | Urease_gamma | C30 |
| Total dissolved nitrogen | Alpha-amylase | C30 |
| Total dissolved nitrogen | DLH | C30 |
| Total dissolved nitrogen | GerE | C18 |
| Total dissolved nitrogen | GTP-bdg_M | C30 |
| Total dissolved nitrogen | OmpH | C37 |
| Total dissolved nitrogen | TANGO2 | C30 |
| C/N ratio | AAA_10 | C49 |
| C/N ratio | ADK_lid | C23 |
| C/N ratio | AIF_C | C13 |
| C/N ratio | Arm | C28 |
| C/N ratio | ATPgrasp_ST | C1 |
| C/N ratio | Bac_DNA_binding | C9 |
| C/N ratio | BofC_C | C15 |
| C/N ratio | BPD_transp_1 | C5 |
| C/N ratio | CpxA_peri | C23 |
| C/N ratio | Cu_amine_oxidN1 | C4 |
| C/N ratio | DNA_pol3_delta | C3 |
| C/N ratio | DUF1294 | C47 |
| C/N ratio | DUF150 | C40 |
| C/N ratio | DUF1659 | C15 |
| C/N ratio | DUF169 | C27 |
| C/N ratio | DUF1863 | C23 |
| C/N ratio | DUF1871 | C23 |
| C/N ratio | DUF1989 | C22 |
| C/N ratio | DUF2867 | C23 |
| C/N ratio | DUF3243 | C28 |
| C/N ratio | DUF3934 | C23 |
| C/N ratio | DUF421 | C28 |
| C/N ratio | DUF4446 | C27 |
| C/N ratio | DUF4921 | C49 |
| C/N ratio | dUTPase_2 | C37 |
| C/N ratio | ERCC3_RAD25_C | C47 |
| C/N ratio | F_bP_aldolase | C9 |
| C/N ratio | FMN_dh | C35 |
| C/N ratio | Fumarate_red_D | C14 |
| C/N ratio | GDE_N | C43 |
| C/N ratio | GerA | C37 |
| C/N ratio | Glyoxalase_2 | C37 |
| C/N ratio | GntR | C4 |
| C/N ratio | Guanylate_kin | C36 |
| C/N ratio | HK | C47 |
| C/N ratio | HTH_30 | C47 |
| C/N ratio | LAB_N | C23 |
| C/N ratio | LUD_dom | C44 |
| C/N ratio | Malic_M | C9 |
| C/N ratio | NADHdh | C9 |
| C/N ratio | NMT1_2 | C36 |
| C/N ratio | OB_aCoA_assoc | C7 |
| C/N ratio | PdxA | C21 |
| C/N ratio | Phage_rep_org_N | C49 |
| C/N ratio | Phos_pyr_kin | C47 |
| C/N ratio | PRD | C15 |
| C/N ratio | PTPS_related | C49 |
| C/N ratio | Reprolysin_2 | C48 |
| C/N ratio | Ribosomal_L28 | C23 |
| C/N ratio | Ribosomal_L31 | C52 |
| C/N ratio | RNaseH_like | C44 |
| C/N ratio | RraB | C13 |
| C/N ratio | RsgI_N | C22 |
| C/N ratio | SDF | C49 |
| C/N ratio | Sigma54_DBD | C49 |
| C/N ratio | SPOB_a | C52 |
| C/N ratio | SpoVR | C30 |
| C/N ratio | Terminase_4 | C30 |
| C/N ratio | TPM_phosphatase | C27 |
| C/N ratio | TrbC | C7 |
| C/N ratio | tRNA_edit | C29 |
| C/N ratio | YjeF_N | C14 |
| C/N ratio | Zincin_1 | C44 |
| Available potassium | AAA_24 | C0 |
| Available potassium | AAA_25 | C49 |
| Available potassium | Arc | C0 |
| Available potassium | Cad | C49 |
| Available potassium | Capsule_synth | C22 |
| Available potassium | Cu2_monoox_C | C22 |
| Available potassium | CVNH | C0 |
| Available potassium | DAHP_synth_2 | C50 |
| Available potassium | DDE_Tnp_1_assoc | C50 |
| Available potassium | DUF1003 | C23 |
| Available potassium | DUF1264 | C35 |
| Available potassium | DUF1273 | C49 |
| Available potassium | DUF2083 | C40 |
| Available potassium | DUF2155 | C0 |
| Available potassium | DUF2177 | C22 |
| Available potassium | DUF2272 | C23 |
| Available potassium | DUF2721 | C23 |
| Available potassium | DUF2852 | C18 |
| Available potassium | DUF3006 | C52 |
| Available potassium | DUF3930 | C23 |
| Available potassium | DUF4203 | C0 |
| Available potassium | DUF442 | C23 |
| Available potassium | Glyco_hydro_76 | C0 |
| Available potassium | Glyco_tran_28_C | C40 |
| Available potassium | GST_N | C6 |
| Available potassium | Hepar_II_III | C23 |
| Available potassium | HNH_5 | C18 |
| Available potassium | HPPK | C40 |
| Available potassium | HTH_Crp_2 | C29 |
| Available potassium | Imm70 | C52 |
| Available potassium | IPT | C50 |
| Available potassium | Lar_restr_allev | C49 |
| Available potassium | M16C_assoc | C52 |
| Available potassium | MazG | C49 |
| Available potassium | MCD | C52 |
| Available potassium | Meth_synt_2 | C49 |
| Available potassium | Methyltransf_18 | C31 |
| Available potassium | NAD_binding_2 | C49 |
| Available potassium | Nitrate_red_gam | C22 |
| Available potassium | Nop | C0 |
| Available potassium | PAS_5 | C0 |
| Available potassium | Patatin | C0 |
| Available potassium | PCYCGC | C50 |
| Available potassium | Pentapeptide | C49 |
| Available potassium | Phage_connector | C49 |
| Available potassium | PHO4 | C23 |
| Available potassium | PNTB | C49 |
| Available potassium | Post_transc_reg | C23 |
| Available potassium | PPC | C22 |
| Available potassium | Prefoldin | C52 |
| Available potassium | Ribosomal_L23 | C49 |
| Available potassium | Ribosomal_L27 | C49 |
| Available potassium | SfsA | C49 |
| Available potassium | Sigma54_AID | C22 |
| Available potassium | SSF | C40 |
| Available potassium | SUFU | C23 |
| Available potassium | SurA_N | C50 |
| Available potassium | Terminase_GpA | C49 |
| Available potassium | Tmemb_cc2 | C50 |
| Available potassium | Tox-HNH-EHHH | C52 |
| Clay content | CbiG_N | C22 |
| Dissolved Fe | 3-HAO | C52 |
| Dissolved Fe | A2M_N_2 | C23 |
| Dissolved Fe | Acyl-CoA_dh_2 | C23 |
| Dissolved Fe | BBP2_2 | C23 |
| Dissolved Fe | CDO_I | C23 |
| Dissolved Fe | Chlorophyllase2 | C17 |
| Dissolved Fe | Chorismate_synt | C23 |
| Dissolved Fe | DUF1178 | C23 |
| Dissolved Fe | DUF1540 | C23 |
| Dissolved Fe | DUF1674 | C47 |
| Dissolved Fe | DUF1874 | C23 |
| Dissolved Fe | DUF190 | C40 |
| Dissolved Fe | DUF1918 | C18 |
| Dissolved Fe | DUF2000 | C23 |
| Dissolved Fe | DUF2201 | C4 |
| Dissolved Fe | DUF3060 | C23 |
| Dissolved Fe | DUF3576 | C23 |
| Dissolved Fe | DUF3911 | C18 |
| Dissolved Fe | DUF4131 | C47 |
| Dissolved Fe | DUF4387 | C18 |
| Dissolved Fe | DUF952 | C23 |
| Dissolved Fe | GTP_CH_N | C23 |
| Dissolved Fe | Head-tail_con | C23 |
| Dissolved Fe | Methyltransf_1N | C23 |
| Dissolved Fe | PAAR_motif | C23 |
| Dissolved Fe | Peptidase_S80 | C23 |
| Dissolved Fe | Phage_AlpA | C23 |
| Dissolved Fe | Phasin_2 | C23 |
| Dissolved Fe | PhnH | C22 |
| Dissolved Fe | PPO1_DWL | C23 |
| Dissolved Fe | Ribosomal_L2_C | C52 |
| Dissolved Fe | SelA | C14 |
| Dissolved Fe | Spo0M | C23 |
| Dissolved Fe | T2SSK | C23 |
| Dissolved Fe | TraD | C23 |
| Dissolved Fe | UDPGP | C22 |
| Dissolved Fe | UPF0093 | C23 |
| Dissolved Fe | Ureidogly_lyase | C23 |
| Dissolved Al | Acyl-CoA_dh_C | C18 |
| Dissolved Al | ADH_N | C43 |
| Dissolved Al | AIG2_2 | C0 |
| Dissolved Al | Alginate_lyase | C18 |
| Dissolved Al | Apocytochr_F_C | C17 |
| Dissolved Al | Archease | C46 |
| Dissolved Al | CobT_C | C18 |
| Dissolved Al | Cu_amine_oxid | C0 |
| Dissolved Al | Cytochrom_C1 | C0 |
| Dissolved Al | DinB | C50 |
| Dissolved Al | DmsC | C23 |
| Dissolved Al | DsbC | C0 |
| Dissolved Al | DUF1127 | C18 |
| Dissolved Al | DUF1285 | C0 |
| Dissolved Al | DUF1929 | C23 |
| Dissolved Al | DUF2235 | C0 |
| Dissolved Al | DUF2877 | C0 |
| Dissolved Al | DUF3048_C | C18 |
| Dissolved Al | DUF3151 | C0 |
| Dissolved Al | DUF3267 | C47 |
| Dissolved Al | DUF3375 | C18 |
| Dissolved Al | DUF3951 | C0 |
| Dissolved Al | DUF411 | C18 |
| Dissolved Al | DUF4169 | C22 |
| Dissolved Al | Gate | C18 |
| Dissolved Al | Glyco_hydro_12 | C0 |
| Dissolved Al | Glyco_hydro_30C | C0 |
| Dissolved Al | HycI | C4 |
| Dissolved Al | Kelch_4 | C17 |
| Dissolved Al | Lin0512_fam | C23 |
| Dissolved Al | LuxS | C16 |
| Dissolved Al | Metalloenzyme | C30 |
| Dissolved Al | Methyltransf_7 | C0 |
| Dissolved Al | MmcB-like | C18 |
| Dissolved Al | oligo_HPY | C0 |
| Dissolved Al | ORF6N | C0 |
| Dissolved Al | PAS | C14 |
| Dissolved Al | Peptidase_S55 | C46 |
| Dissolved Al | Porin_4 | C18 |
| Dissolved Al | Pyr_redox | C44 |
| Dissolved Al | Ribosomal_S19 | C43 |
| Dissolved Al | Ribosomal_S6 | C23 |
| Dissolved Al | RIP | C0 |
| Dissolved Al | RMMBL | C43 |
| Dissolved Al | RNA_pol_Rpb1_1 | C30 |
| Dissolved Al | rRNA_proc-arch | C18 |
| Dissolved Al | RuBisCO_large | C21 |
| Dissolved Al | SBBP | C0 |
| Dissolved Al | Secretin | C23 |
| Dissolved Al | Septum_form | C47 |
| Dissolved Al | Sigma70_r4 | C18 |
| Dissolved Al | SRP54 | C52 |
| Dissolved Al | THDPS_N_2 | C23 |
| Dissolved Al | TK | C30 |
| Dissolved Al | TonB_2 | C0 |
| Dissolved Al | Vut_1 | C14 |
| Dissolved Al | WxL | C27 |
| Dissolved Al | YiiD_C | C23 |
| Dissolved Al | YndJ | C0 |
| Dissolved Al | YuiB | C47 |
| Dissolved Al | YwhD | C9 |

Table S4. The neighbour nodes of functional specific domain of unknown function (DUF) genes.

|  | **DUF genes** | **Pfam ID** | **Pfam accession number** | **Pfam description** |
| --- | --- | --- | --- | --- |
| **1** | DUF2076/DUF4239/DUF1802 | *DUF1802* | PF08819.9 | Domain of unknown function (DUF1802) |
| **2** | DUF2076/DUF4239/DUF1802 | *DUF2076* | PF09849.7 | Uncharacterized protein conserved in bacteria (DUF2076) |
| **3** | DUF2076/DUF4239/DUF1802 | *DUF4239* | PF14023.4 | Protein of unknown function (DUF4239) |
| **4** | DUF2076/DUF4239/DUF1802 | *FAD_binding_3* | PF01494.17 | FAD binding domain |
| **5** | DUF2076/DUF4239/DUF1802 | *Fer4_13* | PF13370.4 | 4Fe-4S single cluster domain of Ferredoxin I |
| **6** | DUF2076/DUF4239/DUF1802 | *FIST* | PF08495.8 | FIST N domain |
| **7** | DUF2076/DUF4239/DUF1802 | *Hydrolase* | PF00702.24 | haloacid dehalogenase-like hydrolase |
| **8** | DUF2076/DUF4239/DUF1802 | *Maf* | PF02545.12 | Maf-like protein |
| **9** | DUF2076/DUF4239/DUF1802 | *PaaA_PaaC* | PF05138.10 | Phenylacetic acid catabolic protein |
| **10** | DUF2076/DUF4239/DUF1802 | *PATR* | PF12951.5 | Passenger-associated-transport-repeat |
| **11** | DUF2076/DUF4239/DUF1802 | *PG_binding_1* | PF01471.16 | Putative peptidoglycan binding domain |
| **12** | DUF2076/DUF4239/DUF1802 | *PTS_IIB* | PF02302.15 | PTS system, Lactose/Cellobiose specific IIB subunit |
| **13** | DUF2076/DUF4239/DUF1802 | *Rad51* | PF08423.9 | Rad51 |
| **14** | DUF2076/DUF4239/DUF1802 | *SmpB* | PF01668.16 | SmpB protein |
| **15** | DUF2076/DUF4239/DUF1802 | *T2SSG* | PF08334.9 | Type II secretion system (T2SS), protein G |
| **16** | DUF2076/DUF4239/DUF1802 | *UPF0054* | PF02130.15 | Uncharacterized protein family UPF0054 |
| **17** | DUF1343/DUF554 | *AzlD* | PF05437.10 | Branched-chain amino acid transport protein (AzlD) |
| **18** | DUF1343/DUF554 | *BBE* | PF08031.10 | Berberine and berberine like |
| **19** | DUF1343/DUF554 | *CAP* | PF00188.24 | Cysteine-rich secretory protein family |
| **20** | DUF1343/DUF554 | *DUF1343* | PF07075.9 | Protein of unknown function (DUF1343) |
| **21** | DUF1343/DUF554 | *DUF554* | PF04474.10 | Protein of unknown function (DUF554) |
| **22** | DUF1343/DUF554 | *Fer2_4* | PF13510.4 | 2Fe-2S iron-sulfur cluster binding domain |
| **23** | DUF1343/DUF554 | *Fer4_10* | PF13237.4 | 4Fe-4S dicluster domain |
| **24** | DUF1343/DUF554 | *Frataxin_Cyay* | PF01491.14 | Frataxin-like domain |
| **25** | DUF1343/DUF554 | *HTH_23* | PF13384.4 | Homeodomain-like domain |
| **26** | DUF1343/DUF554 | *PD40* | PF07676.10 | WD40-like Beta Propeller Repeat |
| **27** | DUF1343/DUF554 | *Peptidase_M41* | PF01434.16 | Peptidase family M41 |
| **28** | DUF1343/DUF554 | *Peptidase_S41* | PF03572.16 | Peptidase family S41 |
| **29** | DUF1343/DUF554 | *Peptidase_U32_C* | PF16325.3 | Peptidase family U32 C-terminal domain |
| **30** | DUF1343/DUF554 | *PfkB* | PF00294.22 | pfkB family carbohydrate kinase |
| **31** | DUF1343/DUF554 | *RadC* | PF04002.13 | RadC-like JAB domain |
| **32** | DUF1343/DUF554 | *Ribosomal_S30AE* | PF02482.17 | Sigma 54 modulation protein / S30EA ribosomal protein |
| **33** | DUF2969/DUF436 | *Amidohydro_1* | PF01979.18 | Amidohydrolase family |
| **34** | DUF2969/DUF436 | *Carboxyl_trans* | PF01039.20 | Carboxyl transferase domain |
| **35** | DUF2969/DUF436 | *Cass2* | PF14526.4 | Integron-associated effector binding protein |
| **36** | DUF2969/DUF436 | *CTP_transf_like* | PF01467.24 | Cytidylyltransferase-like |
| **37** | DUF2969/DUF436 | *DDE_Tnp_ISL3* | PF01610.15 | Transposase |
| **38** | DUF2969/DUF436 | *DisA_N* | PF02457.14 | DisA bacterial checkpoint controller nucleotide-binding |
| **39** | DUF2969/DUF436 | *DUF285* | PF03382.12 | Mycoplasma protein of unknown function, DUF285 |
| **40** | DUF2969/DUF436 | *DUF2969* | PF11184.6 | Protein of unknown function (DUF2969) |
| **41** | DUF2969/DUF436 | *DUF436* | PF04260.10 | Protein of unknown function (DUF436) |
| **42** | DUF2969/DUF436 | *EIIA-man* | PF03610.14 | PTS system fructose IIA component |
| **43** | DUF2969/DUF436 | *FecCD* | PF01032.16 | FecCD transport family |
| **44** | DUF2969/DUF436 | *Glyco_tranf_2_5* | PF13712.4 | Glycosyltransferase like family |
| **45** | DUF2969/DUF436 | *HNH_3* | PF13392.4 | HNH endonuclease |
| **46** | DUF2969/DUF436 | *Pro_isomerase* | PF00160.19 | Cyclophilin type peptidyl-prolyl cis-trans isomerase/CLD |
| **47** | DUF2969/DUF436 | *SBP_bac_3* | PF00497.18 | Bacterial extracellular solute-binding proteins, family 3 |
| **48** | DUF2969/DUF436 | *SecE* | PF00584.18 | SecE/Sec61-gamma subunits of protein translocation complex |
| **49** | DUF2969/DUF436 | *SpoIIIAC* | PF06686.9 | Stage III sporulation protein AC/AD protein family |
| **50** | DUF1054 | *AAA_15* | PF13175.4 | AAA ATPase domain |
| **51** | DUF1054 | *Caa3_CtaG* | PF09678.8 | Cytochrome c oxidase caa3 assembly factor (Caa3_CtaG) |
| **52** | DUF1054 | *CM_2* | PF01817.19 | Chorismate mutase type II |
| **53** | DUF1054 | *DAO_C* | PF16901.3 | C-terminal domain of alpha-glycerophosphate oxidase |
| **54** | DUF1054 | *DUF1054* | PF06335.10 | Protein of unknown function (DUF1054) |
| **55** | DUF1054 | *DUF4004* | PF13171.4 | Protein of unknown function (DUF4004) |
| **56** | DUF1054 | *FtsA* | PF14450.4 | Cell division protein FtsA |
| **57** | DUF1054 | *GST_C* | PF00043.23 | Glutathione S-transferase, C-terminal domain |
| **58** | DUF1054 | *HisKA_2* | PF07568.10 | Histidine kinase |
| **59** | DUF1054 | *K_trans* | PF02705.14 | K+ potassium transporter |
| **60** | DUF1054 | *Na_H_Exchanger* | PF00999.19 | Sodium/hydrogen exchanger family |
| **61** | DUF1054 | *NIF3* | PF01784.16 | NIF3 (NGG1p interacting factor 3) |
| **62** | DUF1054 | *Peptidase_M6* | PF05547.9 | Immune inhibitor A peptidase M6 |
| **63** | DUF1054 | *Pro_dh* | PF01619.16 | Proline dehydrogenase |
| **64** | DUF1054 | *RDD* | PF06271.10 | RDD family |
| **65** | DUF1054 | *TruB_C_2* | PF16198.3 | tRNA pseudouridylate synthase B C-terminal domain |
| **66** | DUF3324 | *AzlC* | PF03591.12 | AzlC protein |
| **67** | DUF3324 | *Dak2* | PF02734.15 | DAK2 domain |
| **68** | DUF3324 | *DUF1073* | PF06381.9 | Protein of unknown function (DUF1073) |
| **69** | DUF3324 | *DUF3324* | PF11797.6 | Protein of unknown function C-terminal (DUF3324) |
| **70** | DUF3324 | *F420_oxidored* | PF03807.15 | NADP oxidoreductase coenzyme F420-dependent |
| **71** | DUF3324 | *Glucosaminidase* | PF01832.18 | Mannosyl-glycoprotein endo-beta-N-acetylglucosaminidase |
| **72** | DUF3324 | *GramPos_pilinBB* | PF16569.3 | Gram-positive pilin backbone subunit 2, Cna-B-like domain |
| **73** | DUF3324 | *Nuc_deoxyrib_tr* | PF05014.13 | Nucleoside 2-deoxyribosyltransferase |
| **74** | DUF3324 | *PC4* | PF02229.14 | Transcriptional Coactivator p15 (PC4) |
| **75** | DUF3324 | *Pectinesterase* | PF01095.17 | Pectinesterase |
| **76** | DUF3324 | *PTS_EIIB* | PF00367.18 | phosphotransferase system, EIIB |
| **77** | DUF3324 | *PurS* | PF02700.12 | Phosphoribosylformylglycinamidine (FGAM) synthase |
| **78** | DUF3324 | *RhaA* | PF06134.9 | L-rhamnose isomerase (RhaA) |
| **79** | DUF3324 | *Ribosomal_L11* | PF00298.17 | Ribosomal protein L11, RNA binding domain |
| **80** | DUF3324 | *Ribosomal_L19* | PF01245.18 | Ribosomal protein L19 |
| **81** | DUF3324 | *Rrf2* | PF02082.18 | Transcriptional regulator |
| **82** | DUF3948 | *AdoMet_dc* | PF02675.13 | S-adenosylmethionine decarboxylase |
| **83** | DUF3948 | *Bac_export_1* | PF01311.18 | Bacterial export proteins, family 1 |
| **84** | DUF3948 | *CppA_C* | PF14507.4 | CppA C-terminal |
| **85** | DUF3948 | *DUF3948* | PF13134.4 | Protein of unknown function (DUF3948) |
| **86** | DUF3948 | *FliG_N* | PF14842.4 | FliG N-terminal domain |
| **87** | DUF3948 | *Ftsk_gamma* | PF09397.8 | Ftsk gamma domain |
| **88** | DUF3948 | *Intg_mem_TP0381* | PF09529.8 | Integral membrane protein (intg_mem_TP0381) |
| **89** | DUF3948 | *Lyase_8_N* | PF08124.9 | Polysaccharide lyase family 8, N terminal alpha-helical domain |
| **90** | DUF3948 | *ORF6C* | PF10552.7 | ORF6C domain |
| **91** | DUF3948 | *PepSY* | PF03413.17 | Peptidase propeptide and YPEB domain |
| **92** | DUF3948 | *Peptidase_S11* | PF00768.18 | D-alanyl-D-alanine carboxypeptidase |
| **93** | DUF3948 | *Phytase-like* | PF13449.4 | Esterase-like activity of phytase |
| **94** | DUF3948 | *Pirin_C* | PF05726.11 | Pirin C-terminal cupin domain |
| **95** | DUF3948 | *RF-1* | PF00472.18 | RF-1 domain |
| **96** | DUF3948 | *Sigma70_r3* | PF04539.14 | Sigma-70 region 3 |
| **97** | DUF3948 | *Spore_GerAC* | PF05504.9 | Spore germination B3/ GerAC like, C-terminal |
| **98** | DUF3948 | *SpoVG* | PF04026.10 | SpoVG |
| **99** | DUF808 | *2Fe-2S_thioredx* | PF01257.17 | Thioredoxin-like [2Fe-2S] ferredoxin |
| **100** | DUF808 | *Asp23* | PF03780.11 | Asp23 family, cell envelope-related function |
| **101** | DUF808 | *CheW* | PF01584.17 | CheW-like domain |
| **102** | DUF808 | *Cu-oxidase* | PF00394.20 | Multicopper oxidase |
| **103** | DUF808 | *DUF3383* | PF11863.6 | Protein of unknown function (DUF3383) |
| **104** | DUF808 | *DUF5117* | PF17148.2 | Domain of unknown function (DUF5117) |
| **105** | DUF808 | *DUF808* | PF05661.10 | Protein of unknown function (DUF808) |
| **106** | DUF808 | *DUF885* | PF05960.9 | Bacterial protein of unknown function (DUF885) |
| **107** | DUF808 | *FTSW_RODA_SPOVE* | PF01098.17 | Cell cycle protein |
| **108** | DUF808 | *Glyoxal_oxid_N* | PF07250.9 | Glyoxal oxidase N-terminus |
| **109** | DUF808 | *LON_substr_bdg* | PF02190.14 | ATP-dependent protease La (LON) substrate-binding domain |
| **110** | DUF808 | *MarC* | PF01914.15 | MarC family integral membrane protein |
| **111** | DUF808 | *PAF-AH_p_II* | PF03403.11 | Platelet-activating factor acetylhydrolase, isoform II |
| **112** | DUF808 | *RskA* | PF10099.7 | Anti-sigma-K factor rskA |
| **113** | DUF808 | *zf-dskA_traR* | PF01258.15 | Prokaryotic dksA/traR C4-type zinc finger |
| **114** | DUF2834 | *BTAD* | PF03704.15 | Bacterial transcriptional activator domain |
| **115** | DUF2834 | *Caud_tail_N* | PF16838.3 | Caudoviral major tail protein N-terminus |
| **116** | DUF2834 | *CopC* | PF04234.10 | CopC domain |
| **117** | DUF2834 | *DNA_primase_S* | PF01896.17 | DNA primase small subunit |
| **118** | DUF2834 | *DUF2834* | PF11196.6 | Protein of unknown function (DUF2834) |
| **119** | DUF2834 | *DUF998* | PF06197.11 | Protein of unknown function (DUF998) |
| **120** | DUF2834 | *EthD* | PF07110.9 | EthD domain |
| **121** | DUF2834 | *FHA* | PF00498.24 | FHA domain |
| **122** | DUF2834 | *Glycolipid_bind* | PF06475.9 | Putative glycolipid-binding |
| **123** | DUF2834 | *Glyco_hydro_15* | PF00723.19 | Glycosyl hydrolases family 15 |
| **124** | DUF2834 | *Glyco_trans_1_2* | PF13524.4 | Glycosyl transferases group 1 |
| **125** | DUF2834 | *Glyco_trans_4_4* | PF13579.4 | Glycosyl transferase 4-like domain |
| **126** | DUF2834 | *LYTB* | PF02401.16 | LytB protein |
| **127** | DUF2834 | *Phytase* | PF02333.13 | Phytase |
| **128** | DUF2834 | *Ribonuc_red_2_N* | PF08471.8 | Class II vitamin B12-dependent ribonucleotide reductase |
| **129** | DUF2834 | *TGT* | PF01702.16 | Queuine tRNA-ribosyltransferase |
| **130** | DUF2834 | *Thiol_cytolysin* | PF01289.17 | Thiol-activated cytolysin |

Table S5. Structure of protein domains in DUF genes modelled by homology modelling with SWISS-MODEL. The functions of template domain structure closely associating with predicted functions of DUF genes were showed in red bold font.

| **Pfam ID** | **Predicted function** | **Length** | **Position**  **(bp)** | **Modeling structure** | **Template ID** | **Template structure** | **Template function** | **Identity** |
| --- | --- | --- | --- | --- | --- | --- | --- | --- |
| **DUF1343** | Protein metabolism | 359 | 16-135 | 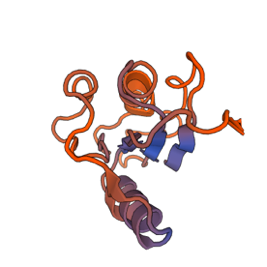 | 4xyl.1.A | 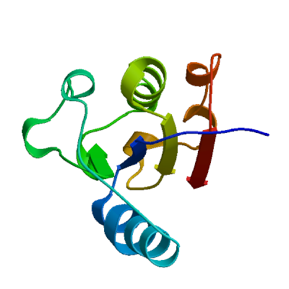 | Alpha-subunit of Acyl-CoA synthetase | 17.39% |
|  |  |  | 28-86 | 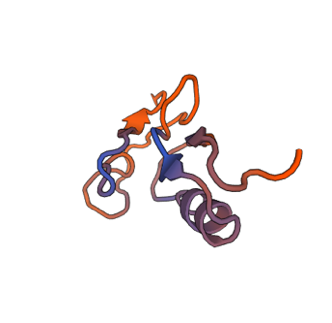 | 2prz.1.A | 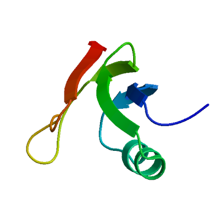 | **Orotate phosphoribosyl-transferase: an enzyme involved in pyrimidine biosynthesis** ^1^**.** | 23.08% |
|  |  |  | 123-141 | 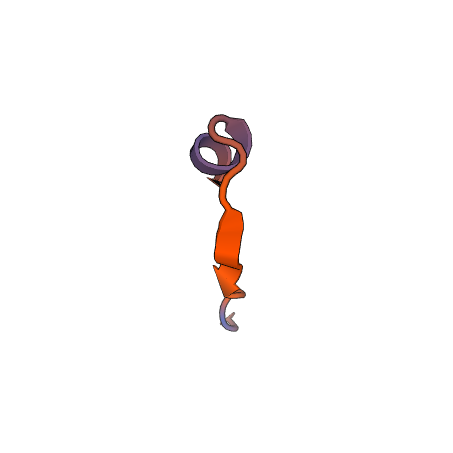 | 4x7g.1.A | 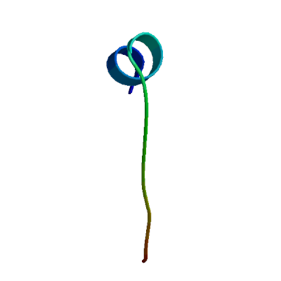 | **Precorrin-6A reductase: this enzyme catalyzes formation of Vitamin B12, which play important roles in protein metabolism** ^2^**.** | 23.81% |
| **DUF2969** | Transferase activity | 68 | 4-40 | 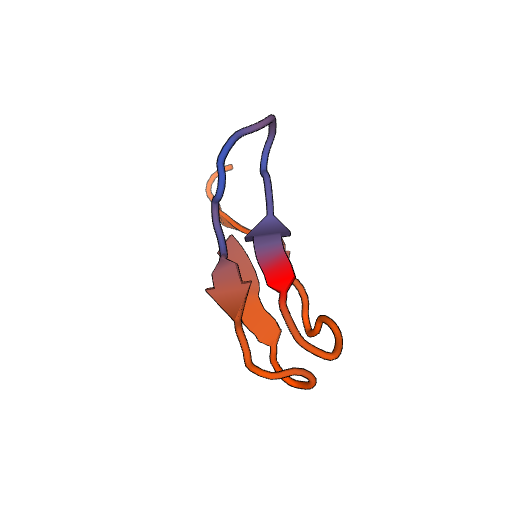 | 5c71.1.A | 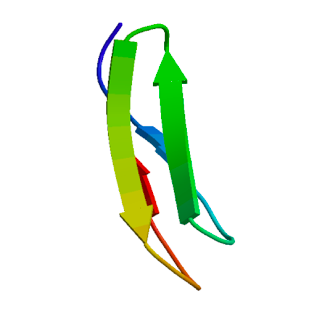 | **Glucuronidase: Beta-glucuronidase is closely associated with UDP-glucuronyltransferase** ^3^**.** | 21.28% |
|  |  |  | 12-64 | 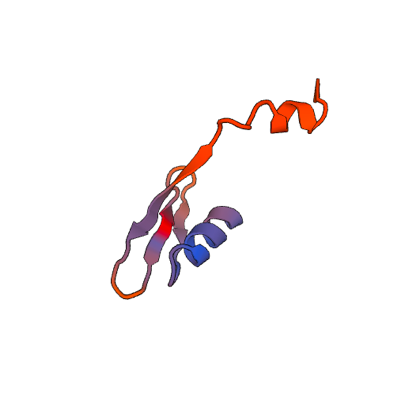 | 3k6q.1.B | 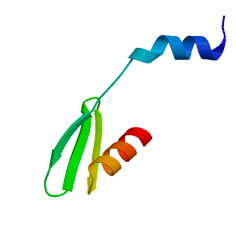 | **Putative ligand binding protein: ligand binding protein could express in *E. coli* as a glutathione-S-transferase fusion protein** ^4^**.** | 21.28% |
|  |  |  | 23-67 | 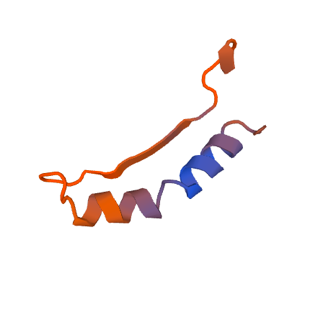 | 4ydu.1.A | 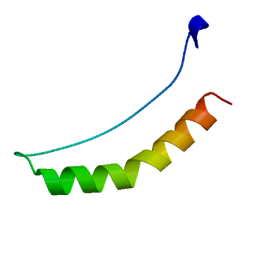 | **tRNA N6-adenosine threonylcarbamoyl- transferase.** | 21.28% |
| **DUF2076** | Organic substance metabolism | 231 | 12-73 | 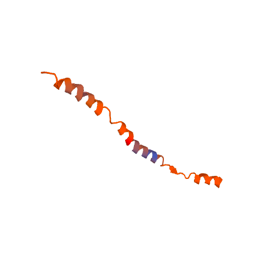 | 3fx0.1.B | 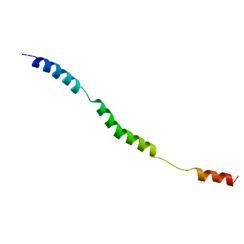 | NF-Kappa_B essential modulator | 26.39% |
|  |  |  | 9-79 | 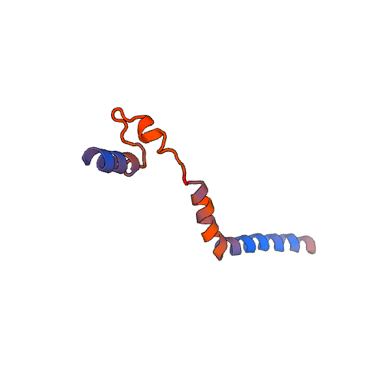 | 5gai.1.W | 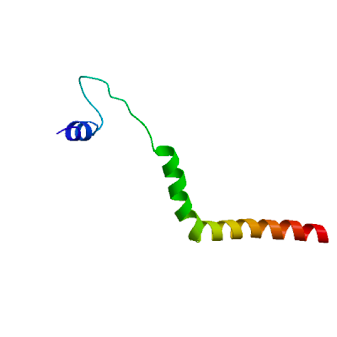 | Portal protein | 17.14% |
|  |  |  | 6-57 | 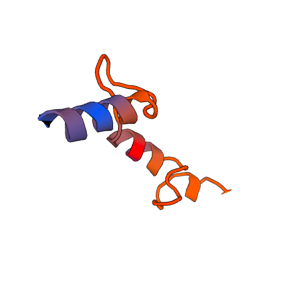 | 4ati.1.A | 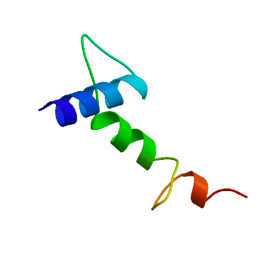 | **Micropthalmia associated transcription factor: this enzyme could regulate metabolism processes in mitochondrion** ^5^**.** | 18.18% |
|  |  |  | 6-78 | 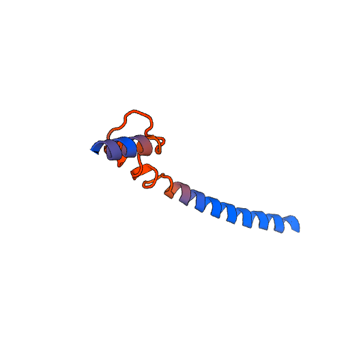 | 5eyo.1.A | 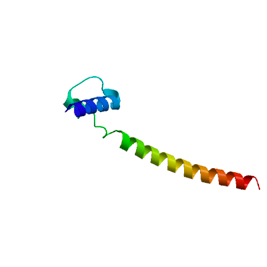 | Protein max | 16.18% |
| **DUF1802** | Organic substance metabolism | 495 | 162-191 | 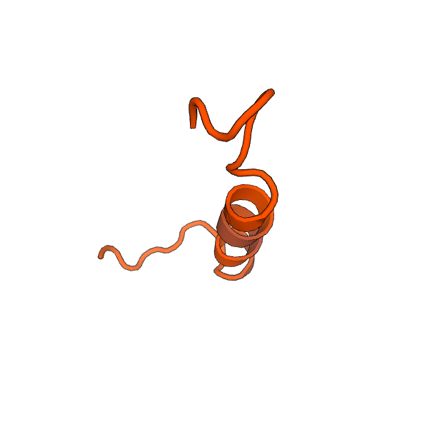 | 1zau.1.A | 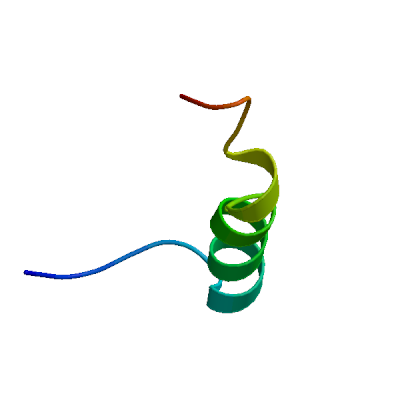 | DNA ligase | 34.48% |
|  |  |  | 248-367 | 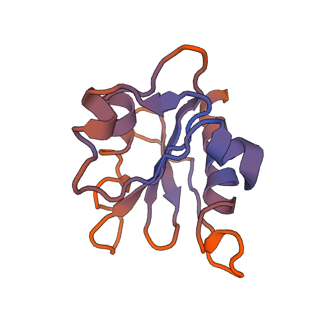 | 4oc8.1.A | 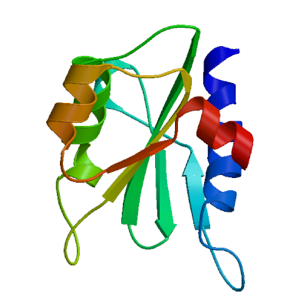 | restriction endonuclease AspBHI | 16.04% |
|  |  |  | 276-360 | 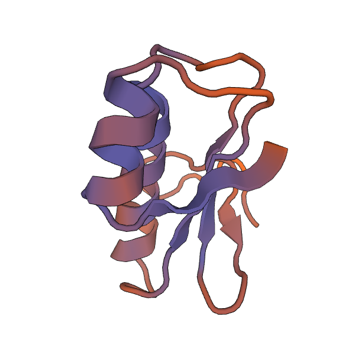 | 5gke.1.A | 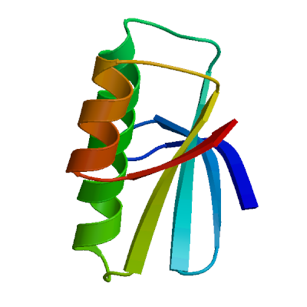 | Endonuclease EndoMS | 20.00% |
|  |  |  | 401-440 | 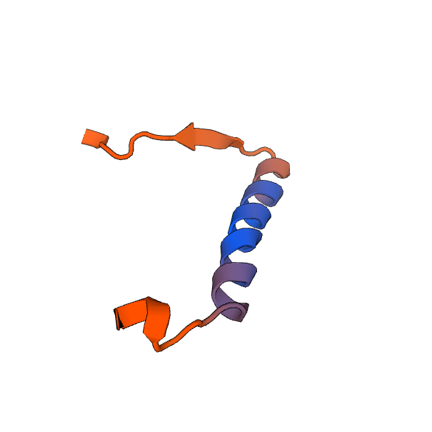 | 4ycw.2.A | 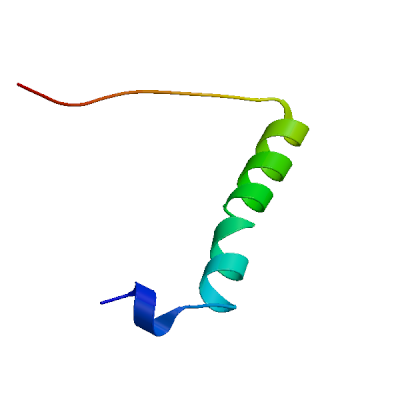 | Lysine--tRNA ligase | 30.00% |
| **DUF4239** | Organic substance metabolism | 265 | 52-244 | 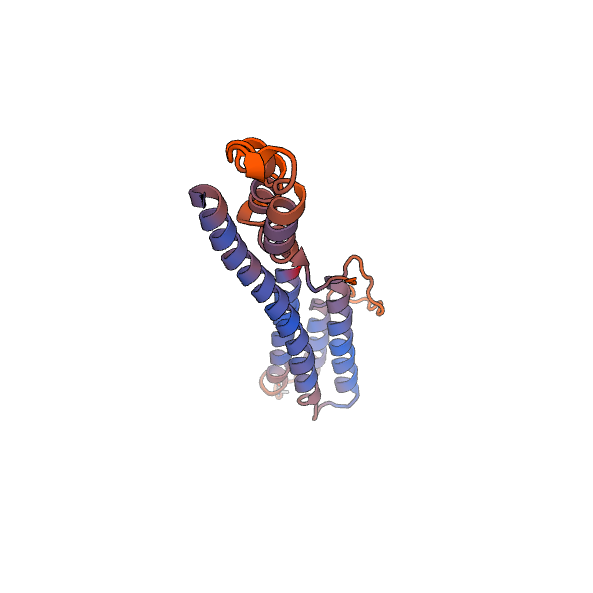 | 4wd8.1.A | 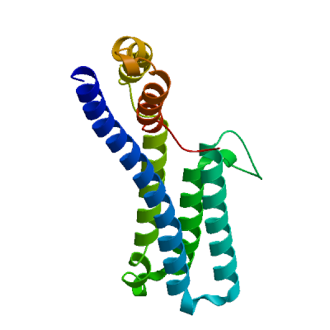 | **Bestrophin domain protein: Bestrophin-2 is a candidate calcium-activated chloride channel** ^6^**, which is potentially involved in the dehalogenation of haloacids.** | 13.37% |
|  |  |  | 78-115 | 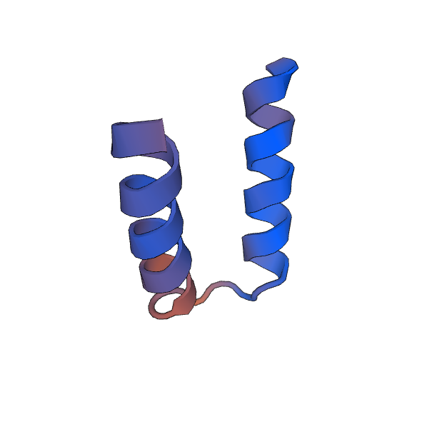 | 3zib.1.A | 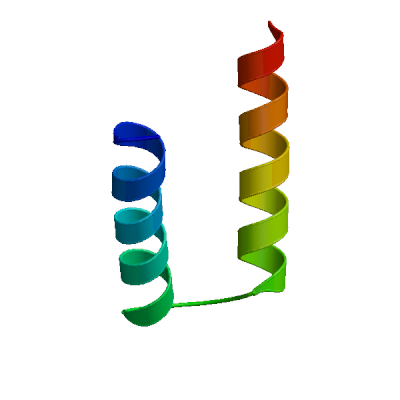 | RAP2A SMA2265 | 13.16% |
|  |  |  | 92-119 | 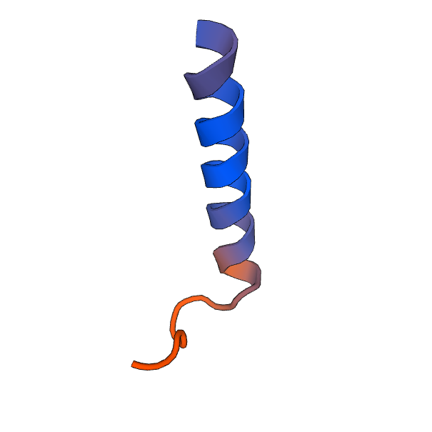 | 4cem.1.A | 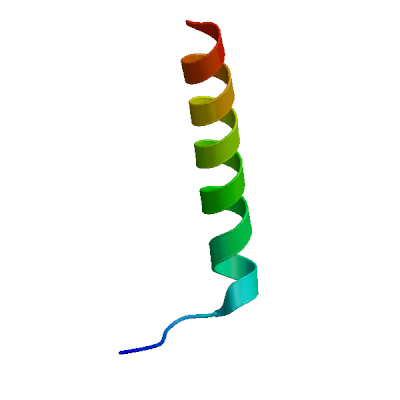 | Regulator of nonsense transcripts 2 | 14.29% |
| **DUF2834** | Glycosyl compound metabolism | 164 | 30-59 | 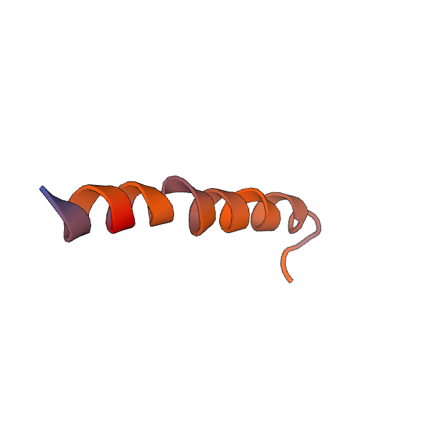 | 3l1l.1.A | 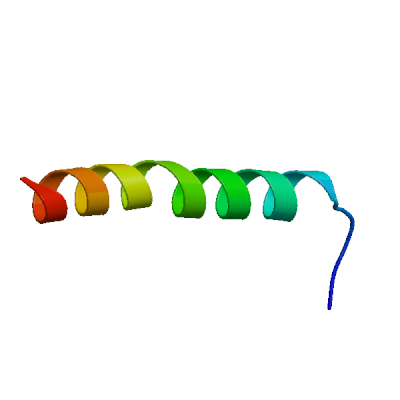 | **Arginine/agmatine antiporter: this enzyme is a transmemberane transporter. Since glycosyl compounds areessential for membrane** ^7^**, Arginine/agmatine antiporter is expected to closely associate with glycosyl compound metabolism.** | 16.67% |
|  |  |  | 120-147 | 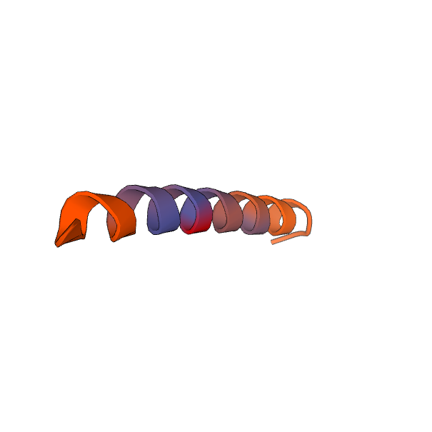 | 5tsa.1.A | 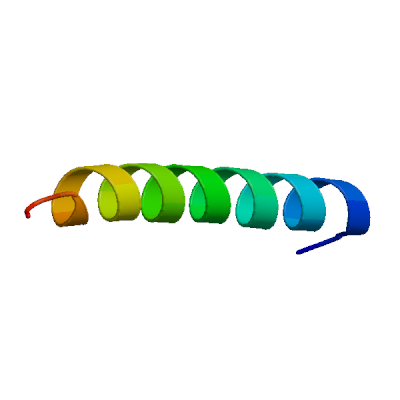 | **Membrane protein: Since glycosyl compounds areessential for membrane** ^8^**, a membrane protein is expected to closely associated with glycosyl compound metabolism.** | 18.52% |
|  |  |  | 129-155 | 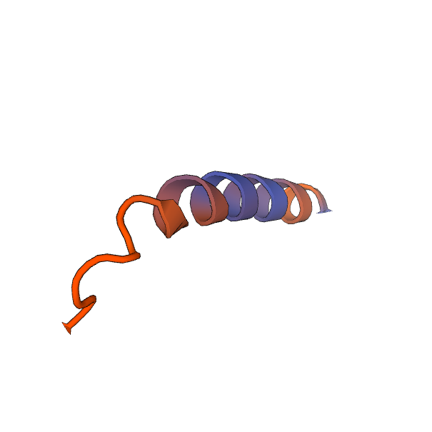 | 2m20.1.A | 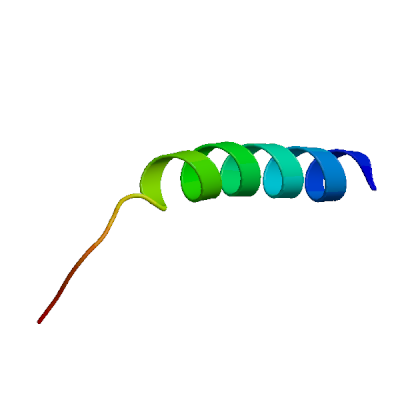 | **Epidermal growth factor receptor: this enzyme is a transmembrane protein as well. Since glycosyl compounds areessential for membrane** ^8^**, epidermal growth factor receptor is expected to closely associated with glycosyl compound metabolism.** | 14.81% |
| **DUF3324** | Transcription process | 341 | 49-280 | 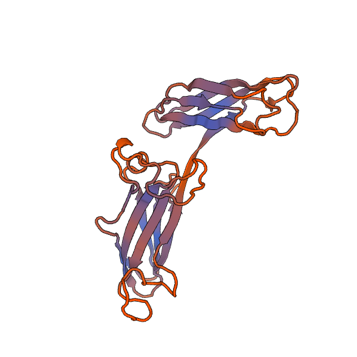 | 1l9m.1.A | 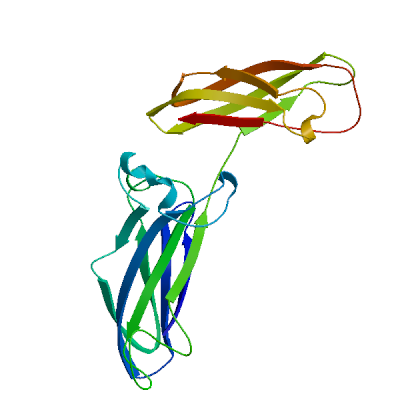 | Protein-glutamine glutamyltransferase E3: | 13.23% |
|  |  |  | 61-276 | 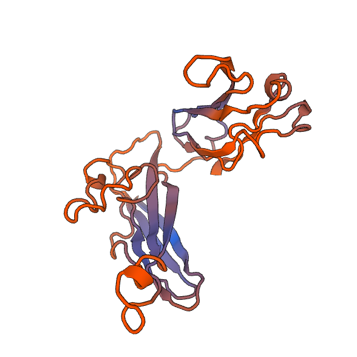 | 3q48.1.A | 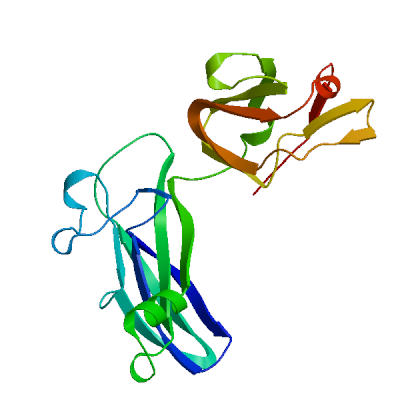 | **Chaperone: Histone chaperone HIRA has reported to regulate transcription factor RUNX1** ^9^**.** | 11.83% |
|  |  |  | 207-309 | 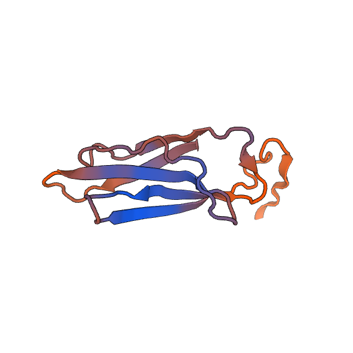 | 3fn9.1.A | 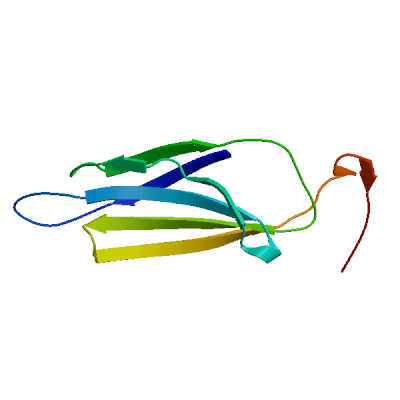 | Putative beta-galactosidase | 14.00% |
|  |  |  | 208-282 | 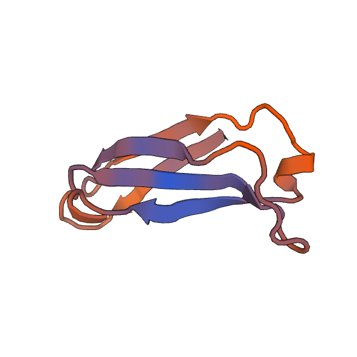 | 2wbk.1.A | 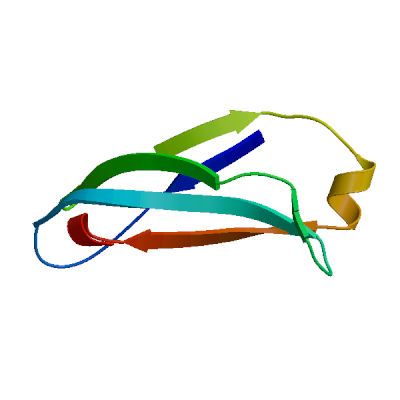 | Beta-mannosidase | 16.22% |
|  |  |  | 207-287 | 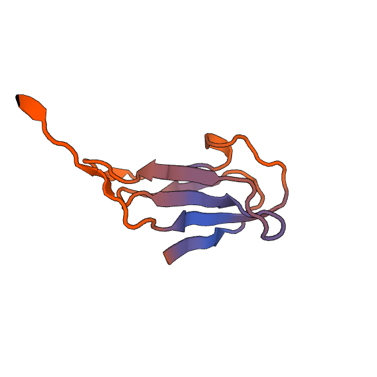 | 3nqh.1.A | 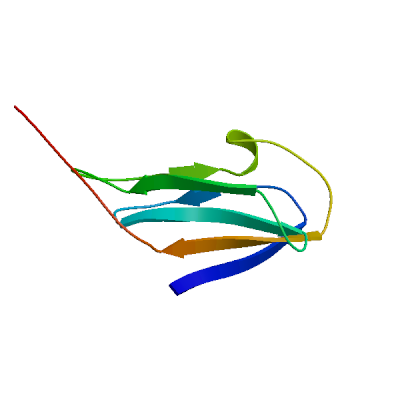 | Glycosyl hydrolase | 16.88% |

**Table S6. Summary of sequencing data**

| **Sample ID** | **Yield (Mbases)** | **Read (M)** | **% ≥ Q30** | **After quality control (M)** |
| --- | --- | --- | --- | --- |
| **BF-1** | 6275 | 41.83 | 93.05 | 5.79 |
| **BF-2** | 6393 | 42.62 | 93.92 | 5.72 |
| **BF-3** | 6039 | 40.26 | 93.75 | 4.69 |
| **BF-4** | 4313 | 28.76 | 93.51 | 4.70 |
| **BF-5** | 5419 | 36.13 | 93.41 | 4.42 |
| **TMCF-1** | 6831 | 45.54 | 93.97 | 3.87 |
| **TMCF-2** | 3330 | 22.20 | 92.88 | 5.71 |
| **TMCF-3** | 4448 | 29.65 | 93.40 | 1.53 |
| **TMCF-4** | 3229 | 21.53 | 92.24 | 5.41 |
| **TMCF-5** | 4783 | 31.88 | 93.19 | 0.67 |
| **TMCF-6** | 6696 | 44.64 | 93.78 | 6.38 |
| **TMCF-7** | 4868 | 32.45 | 93.43 | 2.74 |
| **TMCF-8** | 6569 | 43.79 | 93.34 | 5.23 |
| **TMCF-9** | 6250 | 41.67 | 93.40 | 4.11 |
| **TMCF-10** | 4688 | 31.26 | 92.74 | 2.36 |
| **TDBF-1** | 4334 | 28.89 | 91.68 | 7.40 |
| **TDBF-2** | 5264 | 35.10 | 92.62 | 0.12 |
| **TDBF-3** | 4130 | 27.53 | 92.66 | 2.10 |
| **TDBF-4** | 4747 | 31.65 | 92.89 | 5.83 |
| **TDBF-5** | 7053 | 47.02 | 93.58 | 5.20 |
| **TDBF-6** | 6336 | 42.24 | 93.52 | 11.94 |
| **TDBF-7** | 6010 | 40.07 | 93.03 | 7.32 |
| **TDBF-8** | 6151 | 41.01 | 92.96 | 7.96 |
| **TDBF-9** | 4459 | 29.73 | 93.53 | 5.38 |
| **TDBF-10** | 2175 | 14.50 | 92.06 | 9.45 |
| **SBEF-1** | 5549 | 36.99 | 93.70 | 4.23 |
| **SBEF-2** | 3224 | 21.49 | 92.77 | 9.28 |
| **SBEF-3** | 3176 | 21.17 | 92.90 | 6.31 |
| **SBEF-4** | 3740 | 24.93 | 92.87 | 5.80 |
| **SBEF-5** | 4971 | 33.14 | 92.91 | 9.29 |
| **SBEF-6** | 6094 | 40.63 | 93.60 | 7.28 |
| **SBEF-7** | 5913 | 39.42 | 94.11 | 13.52 |
| **SBEF-8** | 5938 | 39.59 | 93.89 | 11.67 |
| **SBEF-9** | 6736 | 44.91 | 93.93 | 13.84 |
| **SBEF-10** | 3693 | 24.62 | 93.46 | 11.26 |
| **SBEF-11** | 5583 | 37.22 | 93.54 | 10.20 |
| **SBEF-12** | 4946 | 32.97 | 93.23 | 5.17 |
| **SBEF-13** | 5703 | 38.02 | 92.93 | 6.89 |
| **SBEF-14** | 6726 | 44.83 | 93.85 | 8.75 |
| **TSF-1** | 2992 | 19.95 | 92.88 | 8.74 |
| **TSF-2** | 4321 | 28.81 | 92.88 | 4.71 |
| **TSF-3** | 2094 | 13.96 | 91.26 | 5.22 |
| **TSF-4** | 2645 | 17.64 | 91.24 | 10.92 |
| **TSF-5** | 4999 | 33.33 | 93.17 | 7.08 |
| **TSF-6** | 8169 | 54.46 | 93.97 | 8.23 |
